# GBD: incidence rates and prevalence of anxiety disorders, depression and schizophrenia in countries with different SDI levels, 1990–2021

**DOI:** 10.3389/fpubh.2025.1556981

**Published:** 2025-05-16

**Authors:** Jueqi Wang, Xue Guan, Ning Tao

**Affiliations:** School of Public Health, Xinjiang Medical University, Xinjiang, China

**Keywords:** mental disorders, anxiety disorders, depression, schizophrenia, SDI

## Abstract

**Background:**

Anxiety disorders, depression and schizophrenia are the focus of global mental health attention, resulting in a significant number of disability-adjusted life years and a considerable social and economic burden. It’s can affect the socioeconomic landscape as a result of experiencing a global epidemic. And rarely, different Socio-demographic Index (SDI) levels and Age-Period-Cohort (APC) have been used to evaluate the prevalence of mental disorders worldwide.

**Methods:**

Using data from the Global Burden of Disease 2021 (GBD) database, this study assessed trends in the incidence and prevalence of anxiety disorders, depression, and schizophrenia in countries with different SDI levels from 1990 to 2021. Joinpoint and periodic cohort (APC) models were used to sort out the effects of age, period and cohort on incidence. Data were categorized into 5-year age groups and 95% uncertainty intervals (UI) were calculated to account for data variability.

**Results:**

In countries with different SDI levels, the age-standardized average annual percentage change (AAPC) in the incidence of anxiety were all shown to be increasing, and there were large gender differences between the different SDI levels, with a maximum of 0.97 (0.76–1.18) for females in countries with a high SDI level, Age-standardized more rates per 100,000 people in high SDI countries, from 658.87 in 1990 to 841.56 in 2021, and the largest gender differences in countries with a low to moderate SDI level, with AAPCs for males and females of 0.04 (0.04–0.05), 0.86 (0.63–1.09); for depression, only the countries with medium-high SDI levels were statistically significant compared to the countries with medium-low SDI levels, with AAPCs of 0.05 (0.04–0.07), 0.04 (0.04–0.05); for schizophrenia in addition to the AAPCs of the countries with medium-high SDI levels showed an increase of 0.16 (0.13–0.18); the rest decreased.

**Conclusion:**

This study highlights the current status of global incidence and prevalence of mental disorders and examines the complex interactions between the period of onset and cohort of onset of mental disorders using APC modeling, with differences in gender differences in mental disorders in countries with different SDIs, and significant differences in countries with low to medium SDI levels, requiring further exploration of the mechanisms by which socio-economic development influences gender-specific mental health. Countries with different SDI levels have responded to unique trends within their specific socioeconomic, cultural, and historical contexts, suggesting the need for contextualized public health strategies to effectively respond to and manage the incidence and prevalence of mental disorders in these different settings. Prevalence of mental disorders. This points the way to more in-depth future research on treatments and interventions for mental disorders.

## Introduction

According to the 2019 Global Burden of Disease, Injuries, and Risk Factors (GBD) study (1), mental disorders are recognized as a major cause of the global burden of disease (2). Anxiety disorders, depression, and schizophrenia account for a high proportion of disability-adjusted life years (DALY) among all mental disorders (2). This high burden extends across the lifespan by gender (2). More importantly, previous studies have shown that despite the application of many effective interventions, no reduction in global prevalence and burden has been observed since 1990 (1, 3, 4).

“Mental health is a fundamental human right and essential to the development of all countries” (2). The Lancet Commission on Psychology calls for greater investment in mental health services as part of universal health coverage and better integration of these services into global responses to other health priorities. In order to better serve public mental health, it is important to gain a deeper understanding of the scale of the impact of these mental illnesses, including their distributed populations, the health burdens they impose, and their broader health consequence (5).

The Global Burden of Diseases, Injuries and Risk Factors (GBD) study is an ongoing multi-country research project quantifying health losses worldwide. GBD collects all available data and applies statistical and epidemiological modeling to produce comparable, consistent and comprehensive estimates of the burden of disease by age, sex, geographic location and year. Previous GBD studies have shown that the number of people with anxiety disorders globally has increased from 311 million in 1990 to 458 million in 2019 (a 12.6% increase in age-standardized prevalence), with new diagnoses growing from 31.13 million to 45.82 million (6); the total number of people with depression has risen from 172 million to 279 million, with an increase in disability-adjusted life years (DALYs) by 49.3% (7). Schizophrenia prevalence rose from 131,000 (95% UI: 11.6–14.8) million cases in 1990 to 20.9 (95% UI: 18.5–23.4) million cases in 2016 (8). Studies have shown that mental disorders are related to the economic level of the country, and that the prevalence of anxiety disorders tends to be higher in low-income and middle-income countries compared to high-income countries (9), Sub-Saharan Africa, North Africa and the Middle East, and North Africa and the Middle East have higher prevalence rates of depression, with the exception of Oceania, Tropical Latin America, and high-income North America (1), and the coverage of treatment for schizophrenia shows a significant SDI gradient difference: 82 per cent in high SDI countries compared to only 19 per cent in low SDI countries, resulting in a 10.8 year reduction in the average life expectancy of patients in the latter (1).

However, with the changes in mental illness in the wake of the 2019 global epidemic and the fact that much of the previous research has focussed on differences between continents, with little information on country-level differences, the Socio-Social Demographic Index (SDI) was used, which is a common measure used to determine at which stage of development a country or other geographic area is at. The SDI is scored on a scale of 0 to 1, and is a composite average of the per capita income, average educational attainment, and fertility rankings of all regions in the GBD study. The SDI reflects the socioeconomic and demographic characteristics of a region, and has been shown to correlate with the burden of anxiety disorders, depression, and schizophrenia (3, 8, 10). The implementation of rational and effective preventive interventions can reduce the economic burden of mental health by 23–41 per cent in areas where there are significant social developmental differences (2).

This study fills a significant gap in mental health research by integrating SDI (Socio-Demographic Index) stratification and Age-Period-Cohort (APC) modeling to provide country-level analyses of social developmental trajectories and long-term mental health trends while controlling for cohort confounding effects (11, 12). By decomposing age, period, and cohort influences, we provide a standardized framework to enhance cross-population comparability and mitigate the time bias inherent in single cohort designs (13, 14). By employing joinpoint regression modeling models, this study will describe the ongoing changes in the burden of anxiety, depression and schizophrenia. These insights are intended to increase global awareness of anxiety, depression and schizophrenia and support the design of targeted prevention and intervention strategies based on the specific needs of different regions.

## Method

### Data sources

Incidence and prevalence data for anxiety disorders, depression, and schizophrenia were obtained from the Global Burden of Disease 2021 study, which is available to the public (15). The GBD 2021 study estimated the burden of the majority of diseases and their risk factors for most countries and regions of the world for the years 1990 to 2021. The raw data for mental disorders in the GBD 2021 were mainly obtained from electronic databases (including PsycINFO, PubMed, and Embase) of published literature, supplemented by gray literature and expert consultation (16).

Anxiety, depression, and schizophrenia are included in the GBD psychiatric disorders studied in this paper, and the GBD data, in order to be comparable in measurements, case definitions primarily follow the DSM-IV-TR or ICD-10 criteria as these are used in the majority of included mental health surveys.

The Socio-demographic Index (SDI) is a composite indicator developed by the Global Burden of Disease (GBD) team to provide a comprehensive measure of socio-economic development in a country or region. Its core objective is to provide a standardized stratification of social development for burden of disease analyses, thereby revealing systematic associations between health problems and socio-economic factors. In contrast to other existing socio-economic measures, the SDI introduces fertility to capture the stage of the demographic transition and integrates the economy, education and population in a multidimensional way.

Since population data is required for the APC model analysis and there is no population data for different SDI strata in the GBD database, we chose five countries with different SDI levels as representatives. For example, Australia is a typical developed English-speaking country with a mature social security system and a standardized mental health service network (17); Argentina is a representative of middle-income countries in Latin America, experiencing economic fluctuations but maintaining high coverage of basic education (18); Brazil has the highest level of social inequality among the BRICS countries, reflecting the characteristics of social pressures during the transition period (19); and Angola is a resource-based economy in Africa, where mental health investment only 2.1% of the health budget (20); Haiti, a fragile Caribbean country with a psychiatrist density of 0.03/100,000 people (21). Such a sampling selection satisfies the SDI gradient coverage, with different geographical locations and different types of development represented. The high SDI country is Australia; the high-middle SDI country is Argentina; the medium SDI country is Brazil; the low-middle SDI country is Angola; and the low SDI country is Haiti.

We obtained global incidence and prevalence, age-specific incidence and age-specific prevalence of anxiety disorders, depression and schizophrenia for the period 1990–2021. Age-standardized incidence and age-standardized prevalence data, especially for different SDI levels. It’s possible to import SDI values and classifications directly from the GBD database ‘https://vizhub.healthdata.org/gbd-results/’.

### Statistical analysis

To reflect trends in the burden of anxiety disorders, depression, and schizophrenia worldwide from 1990 to 2021, incidence and prevalence rates were calculated, and the results are reported as 95% uncertainty intervals (UIs), which indicate ranges that may contain the true value with a 95% probability, taking into account various sources of uncertainty in the estimation process. SDI types were downloaded directly from the GBD database as low, medium-low, medium, medium-high, and high to compare the age-standardized incidence and standardized prevalence of anxiety disorders, depression, and schizophrenia by historical period, gender, and location in order to avoid differences in the age composition of the population, and Joinpoint trend analyses were carried out to identify significant changes in temporal trends over time. Because demographic data are required for the APC model to perform analyses, countries with representative levels of SDI were selected as Australia, Argentina, Brazil, Angola, and Haiti. A threshold of *α* = 0.05 was used to ensure statistical significance. Software R 4.33 and JOINPOINT software were used.

### Joinpoint regression analysis

It was used to assess trends over time in the burden of disease due to anxiety disorders, depression and schizophrenia. The model is calculated by estimating the pattern of change in prevalence using the least squares method, avoiding the lack of objectivity of traditional trend analysis based on linear trends. The sum of squares of the residuals between the estimated and actual values is calculated to derive the turning point of the moving trend.

### Age-period-cohort analysis

Intrinsic estimator (IE) methods associated with APC models are applied to address the parameter uncertainty inherent in the age, stage, and cohort effects of the APC model (22). The GBD divides the population into groups of people under 5 years of age and over 95 years of age, and for the purposes of APC model fitting, the age groups are defined as <5, 5–9, 10–14… 0.95+. For a period of 5 years (1992–1996, 1997–2001 … 2017–2021), the total number of prevalences was calculated, as well as the cumulative prevalence of each age group. The APC model picks Cross Age, Period RR, Cohort RR, and calculates Net Drift and Wald Tests.

## Results

**Figure 1:**
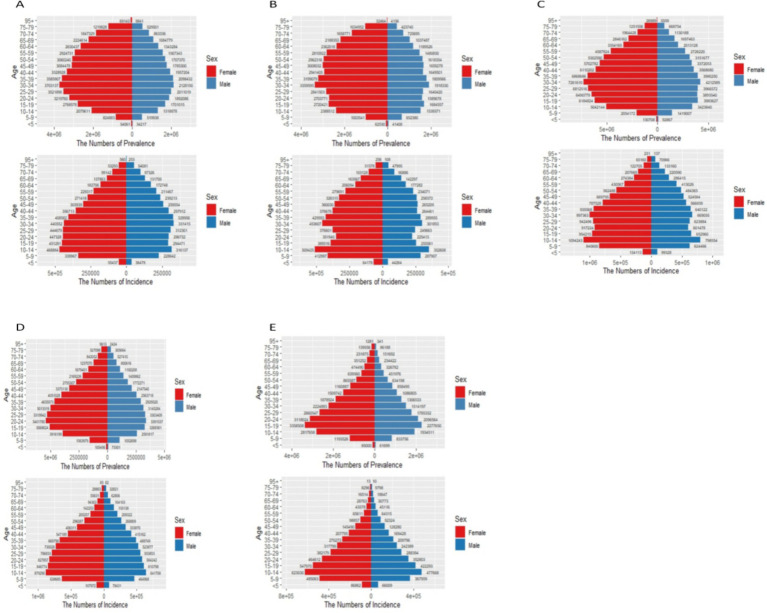
Status of anxiety disorders in countries with different SDI levels in 2021. **(A)** High SDI; **(B)** High-middle SDI; **(C)** Middle SDI; **(D)** Low-middle SDI; **(E)** Low SDI. In the x-axis $\mathrm{ye}+x=y\times 10^{x}$ .

**Figure 2:**
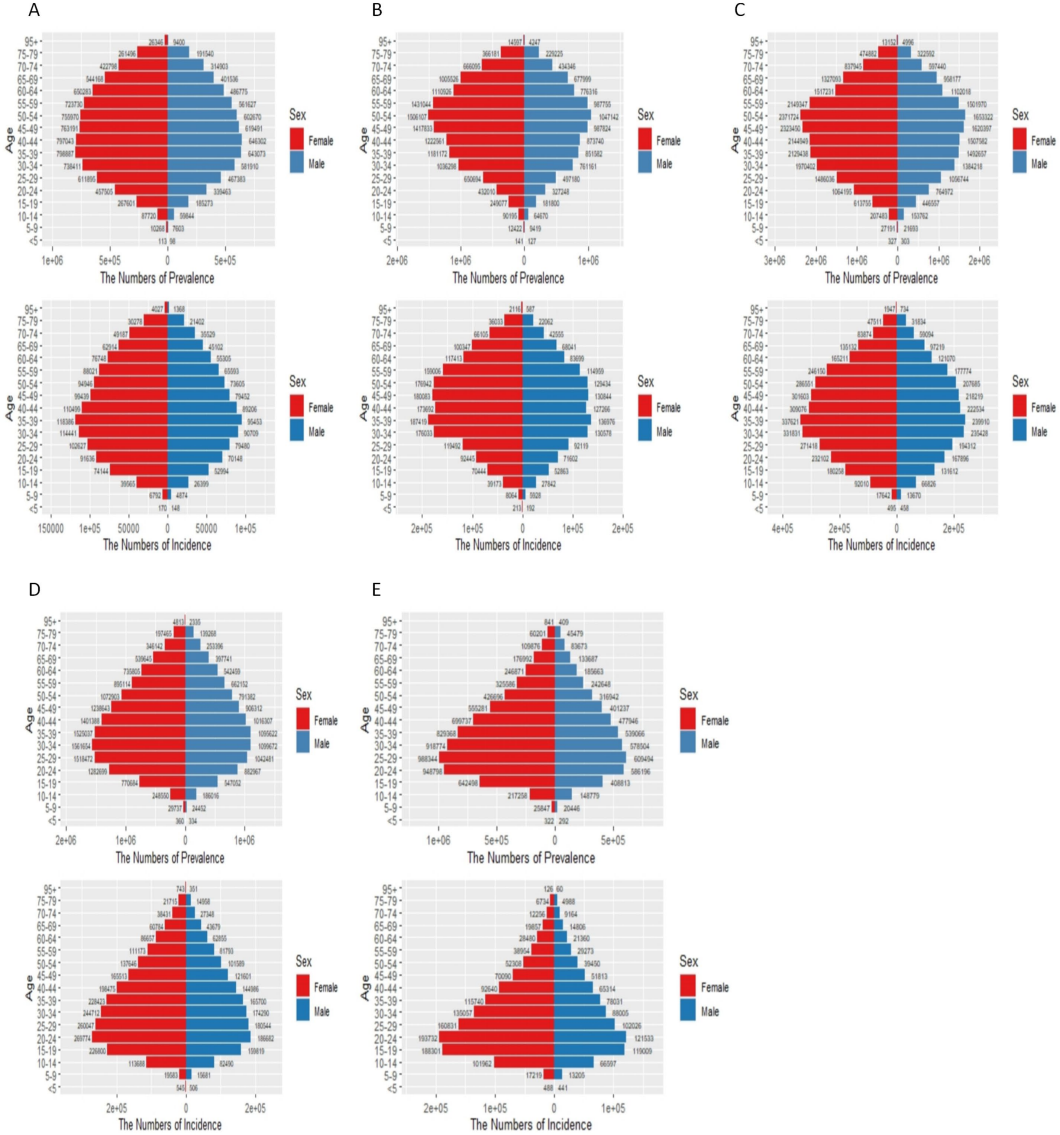
Status of depression in countries with different SDI levels in 2021. **(A)** High SDI; **(B)** High-middle SDI; **(C)** Middle SDI; **(D)** Low-middle SDI; **(E)** Low SDI. In the x-axis $\mathrm{ye}+x=y\times 10^{x}$ .

**Figure 3:**
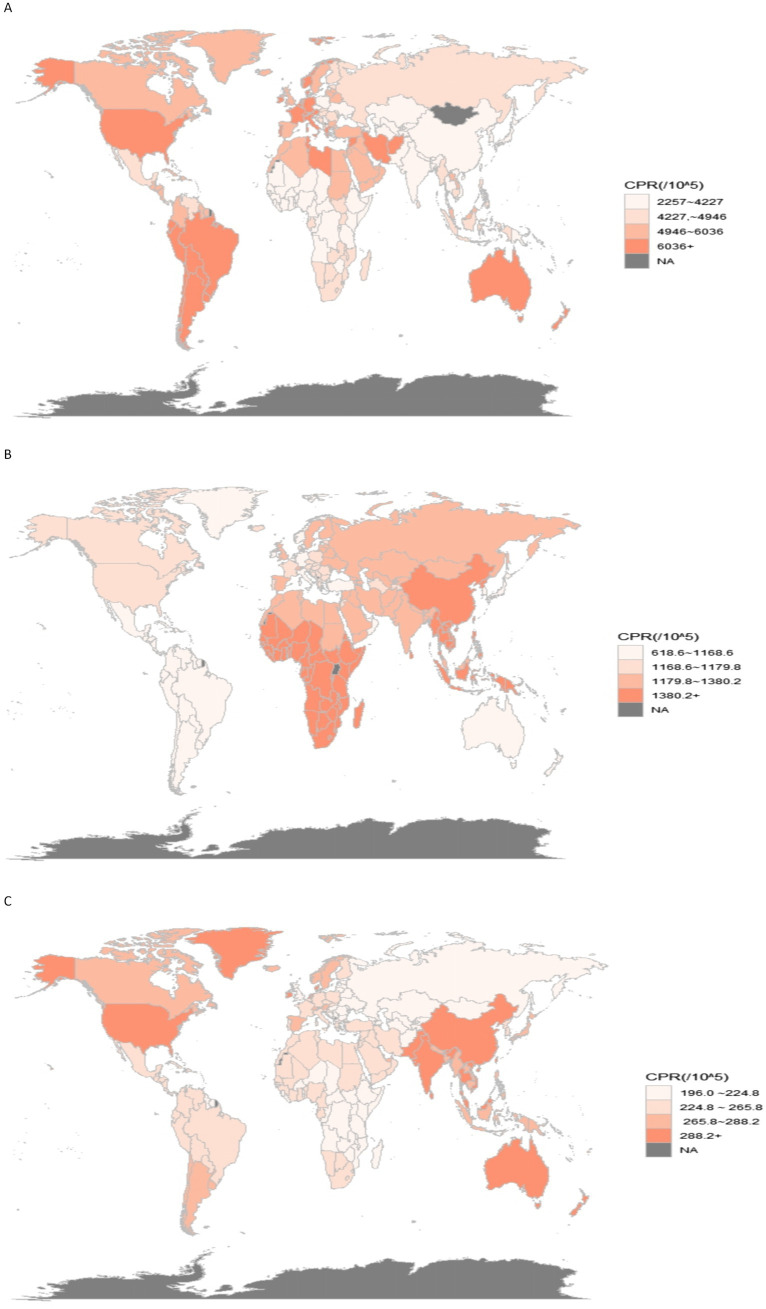
World prevalence of mental disorders 2021. **(A)** Anxiety disorders; **(B)** Depression; **(C)** Schizophrenia.

**Figure 4:**
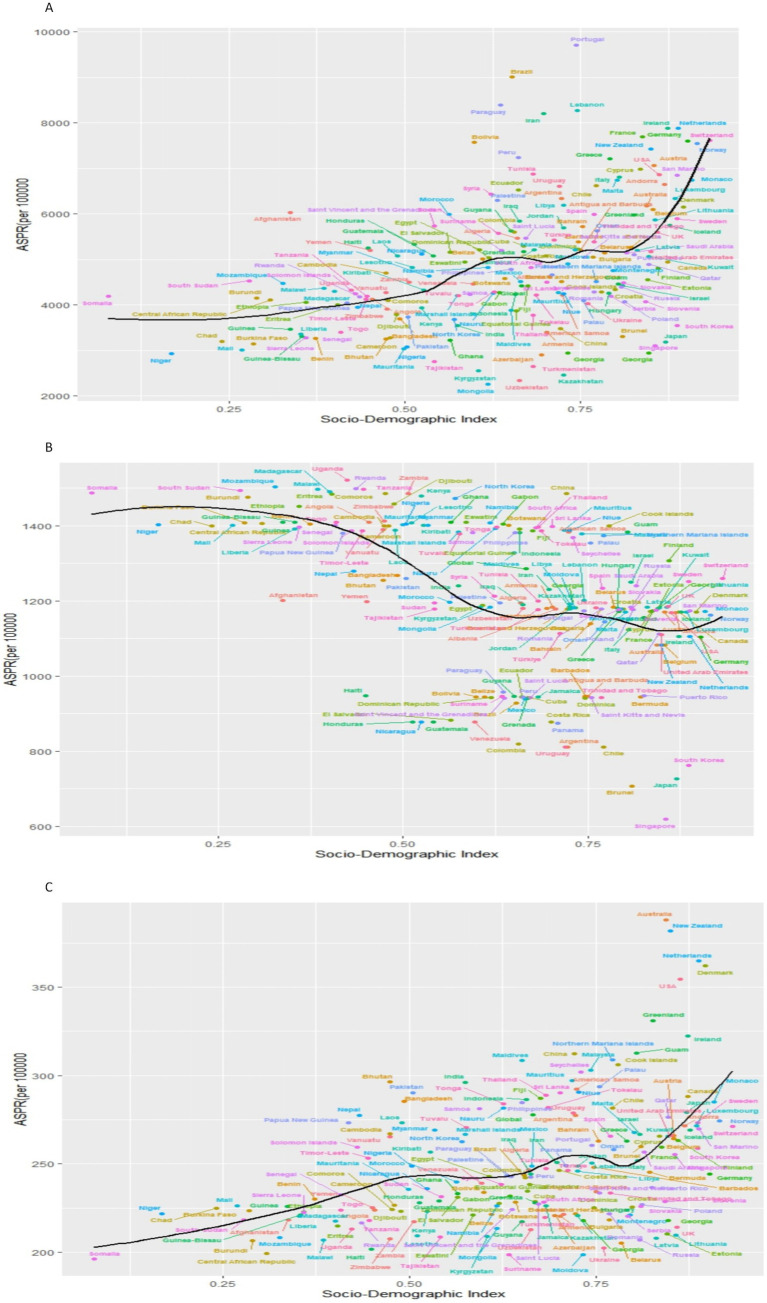
Prevalence of psychiatric disorders at different SDI levels in 2021. **(A)** Anxiety disorders;**(B)** Depression; **(C)** Schizophrenia.

**Figure 5:**
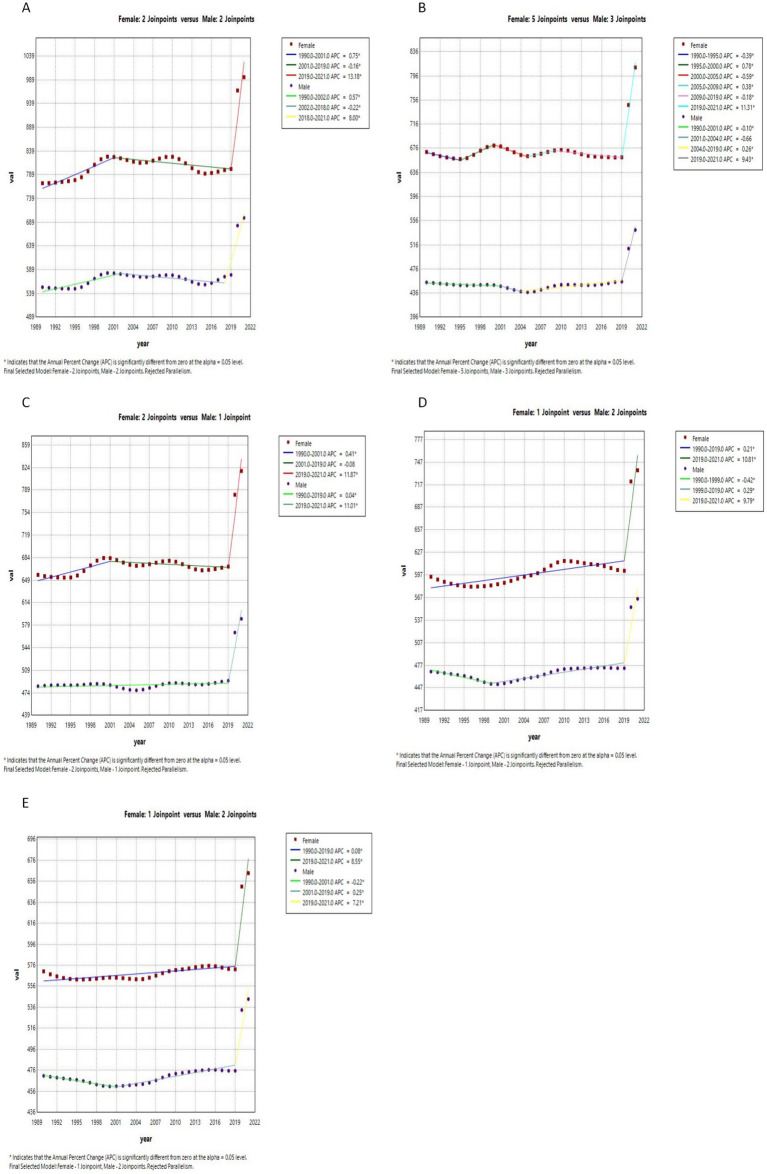
Joinpoints of anxiety disorders at different SDI levels, 1990 to 2021. **(A)** High SDI; **(B)** High-middle SDI; **(C)** Middle SDI; **(D)** Low-middle SDI; **(E)** Low SDI.

Figures 1, 2 in the accompanying table find a sudden spike in the incidence and prevalence of anxiety disorders globally after 2019, with higher prevalence of anxiety disorders in countries with high and medium SDI, lowest prevalence of anxiety disorders in countries with low and medium SDI, and highest prevalence of anxiety disorders in females in countries with high SDI. In Figure 1, it was found that the global prevalence of anxiety disorders is dominated by adolescents aged 10–14 years, with higher prevalence in females than in males, and that the prevalence of anxiety disorders peaks at ages 15–25 years in countries with low and low-moderate SDI levels but at ages 30–35 years in countries with high, high-moderate, and medium SDI levels.13 In the same figure, it was found that the prevalence of anxiety disorders is higher in countries with low and low SDI levels than in countries with high, medium, and medium SDI levels. Figure 3 and Supplementary Tables 13, 14 show that in 2011–2021, anxiety disorders are more prevalent in South America, North America, Europe, and Oceania, and less prevalent in Asia and Africa, as shown in the 2021 Age-standardized for Switzerland, the United States, and China, respectively, 7609.41 (95% CI: 5619.15, 10306.74), 6865.24 (95% CI: 10306.74), and 6865.24 (95% CI: 10306.74), and 6865.24 (95% CI: 10306.74). 6865.24 (95% CI: 5935.69, 7971.2), 3481.74 (95% CI: 2976.2, 4044.48) respectively. According to Figure 4, the number of people with anxiety disorders per 100,000 population increased with increasing SDI levels, with greater increases in countries with high SDI levels. Figure 5 and Supplementary Table 1 show that the difference in trends in anxiety prevalence between males and females is not statistically significant in countries with high SDI values, whereas in countries with SDI values at other levels, the overall trends between the sexes are not parallel, with females experiencing a greater annualized percentage change (APC) in the prevalence of anxiety, with a more rapid rate of increase. Estimates of the APC effect derived from the APC model for different countries are shown in Supplementary Figures 7–9. After adjusting for period effects, the prevalence of anxiety disorders resembles an ‘inverted U-shaped’ curve, with the exception of Angola, where the prevalence peaks at ages 25–30 years, and the rest of the countries at ages 45–50 years, and the period effects in the APC model show an upward trend in the prevalence of anxiety in each of the countries in the 1992–2021 period after 2015, suggesting that the prevalence increases over time. an upward trend, indicating an increased risk of anxiety prevalence over time. Supplementary Figures 10–12 depict trends in the prevalence of psychiatric disorders in different age groups over the period 1992–2021, with the age of peak prevalence of anxiety disorders consistent with that derived from the APC model.

**Figure 6:**
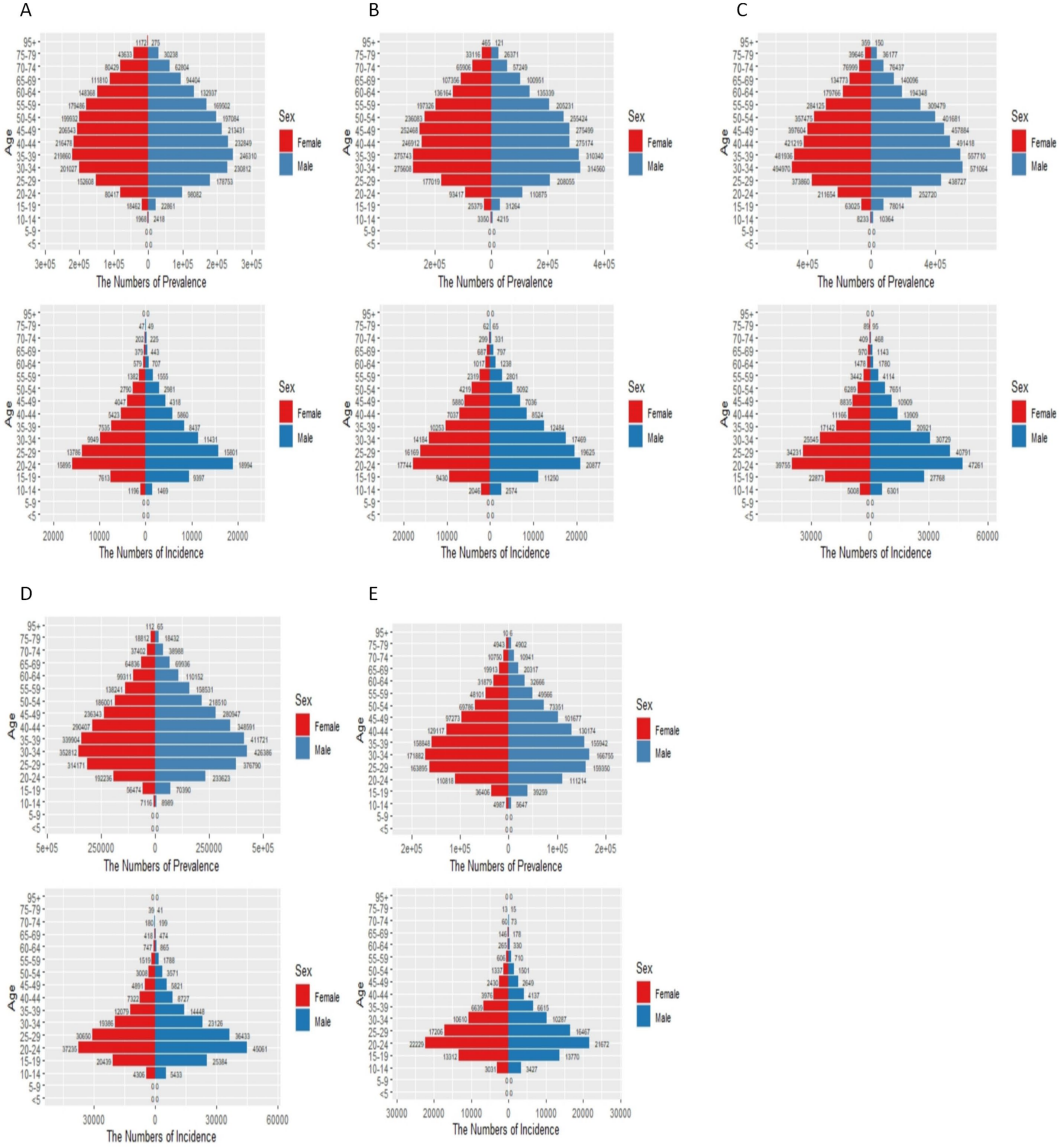
Status of schizophrenia in countries with different SDI levels in 2021. **(A)** High SDI; **(B)** High-middle SDI; **(C)** Middle SDI; **(D)** Low-middle SDI; **(E)** Low SDI. In the x-axis $\mathrm{ye}+x=y\times 10^{x}$ .

**Figure 7:**
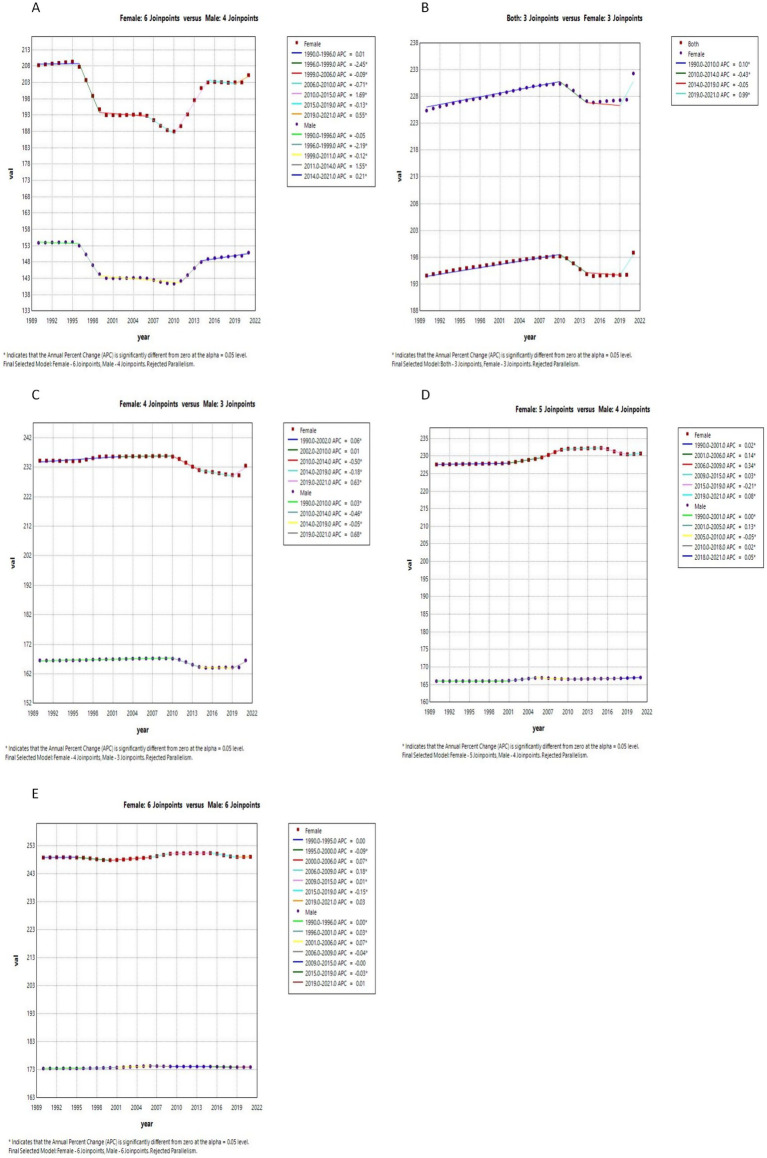
Joinpoints of depression at different SDI levels, 1990 to 2021. **(A)** High SDI; **(B)** High-middle SDI; **(C)** Middle SDI; **(D)** Low-middle SDI; **(E)** Low SDI.

Figures 3, 6 in the accompanying table show that the incidence and prevalence of depression is on the rise in countries with SDI levels other than high SDI countries. Figure 2 shows that the incidence and prevalence of depression is higher among females than males, and that depression peaks at ages 30–39 in countries with high, medium-high, and medium-low SDI levels, while it peaks at ages 20–29 in countries with low, medium-low SDI levels. Figure 3 and Supplementary Tables 13, 14 show that between 2011 and 2021, depression was more prevalent in Asia and Africa and less prevalent in South America and Oceania, as shown by the Age-standardized rates in China, Kenya and Brazil of 1484.98 (95% CI: 1259.76, 1754.51), 1478.46 (95% CI: 1249.76, 1754.51), and 1478.46 (95% CI: 1249.46), respectively, for the period 2021 (95% CI: 1249.84, 1768.34), 927.78 (95% CI: 782.64, 1110.57). According to Figure 4, it shows that the number of people with depression per 100,000 population is higher in countries with low SDI levels and low to medium SDI levels, and that depression decreases with increasing SDI levels. Figure 7 and Supplementary Table 2 show that the difference in trends in depression prevalence between males and females is not statistically significant in countries with low and medium-high SDI levels, whereas the overall trends between the sexes are not parallel in countries with other SDI levels. From 2010 to 2015, there was a significant upward trend in the incidence of depression in high SDI countries, but a downward or flat trend in countries with other SDI levels, and a downward trend in the average annual percentage change (APC) in depression from 1996 to 1999 in high SDI countries. Supplementary Figures 7–9 show estimates of the APC effect from the APC model for different countries, where the prevalence of depression resembles a ‘wavy’ curve, with the peak of prevalence in each country being in the 40s and 50s. The period effect of depression in the APC model shows a decreasing trend in Argentina, where the prevalence of depression has been effectively controlled over time, while Brazil and Angola have shown a decreasing trend. is effectively controlled, while Brazil and Angola show an increasing trend with an increased risk of depression. Supplementary Figures 10–12 depict trends in the prevalence of mental disorders in different age groups over the period 1992–2021, with the age of peak depression consistent with that derived from the APC model.

**Figure 8:**
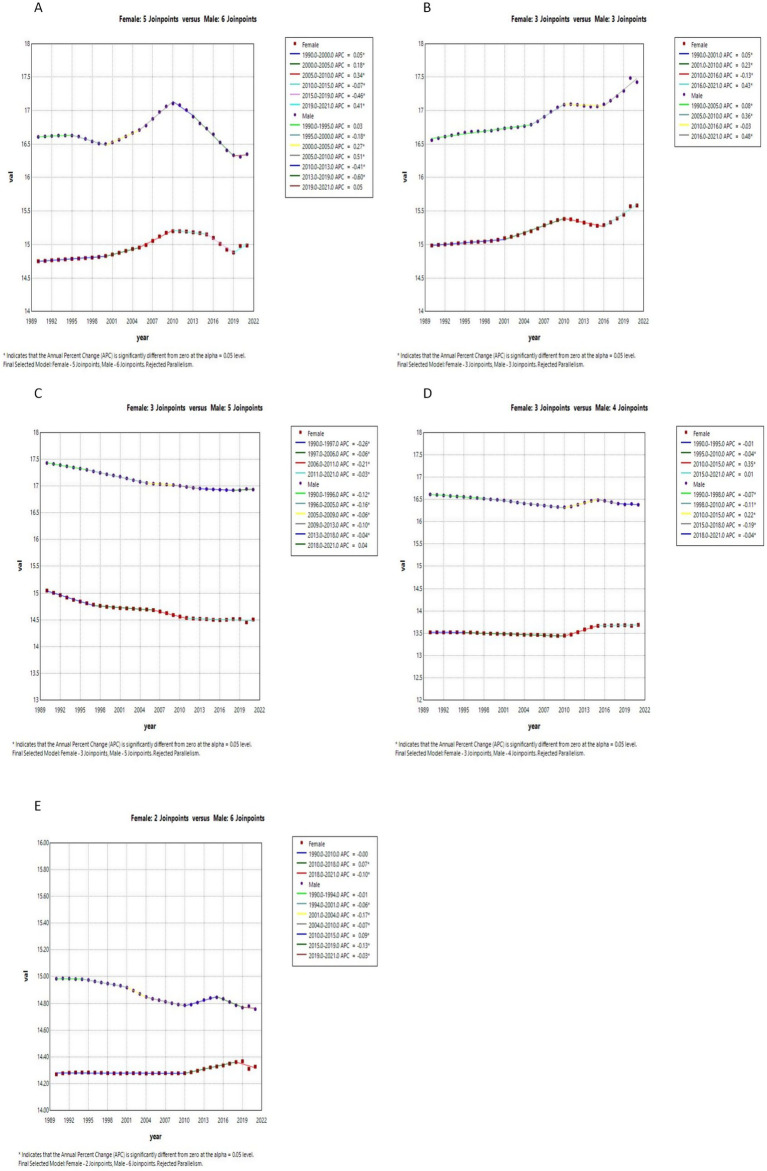
Joinpoints of schizophrenia at different SDI levels, 1990 to 2021. **(A)** High SDI; **(B)** High-middle SDI; **(C)** Middle SDI; **(D)** Low-middle SDI; **(E)** Low SDI.

Figures 4, 5 in the accompanying table show a decreasing trend in the prevalence of schizophrenia symptoms in countries with high SDI, while the number of people with schizophrenia in countries with other SDI levels, although stable in prevalence, is increasing due to population growth. Globally, the highest prevalence of schizophrenia is found in the 20–24 age group, as shown in Figure 6. The number of people diagnosed with schizophrenia drops sharply after the age of 40 years in countries with low, medium, and low to moderate levels of SDI. Figure 3 and Supplementary Tables 13, 14 show that between 2011 and 2021, schizophrenia is more common in Asia, North America and Oceania and less common in Africa. For example, in 2021, China, the United States and the Central African Republic were Age-standardized at 312.36 (95% CI: 271.69, 356.39), 354.43 (95% CI: 298.14, 415.84) and 199.29 (95% CI: 154.55, 254.65), respectively. According to Figure 4, the number of people with schizophrenia per 100,000 population increased with increasing SDI levels, with greater increases in countries with high SDI levels. In Figure 8 and Supplementary Table 3, schizophrenia showed a decreasing trend in all countries except for countries with high SDI levels, which showed an increasing trend, and the average annual percentage change was higher for males than for females. Supplementary Figures 7–9 show the APC effect estimates from the APC model for different countries, with the prevalence of schizophrenia resembling an ‘inverted U-shaped’ curve with no prevalence in the 0–10 age group, and with a peak in prevalence in each country in the 35–45 year age range. Upward trend, while Australia and Argentina remain relatively consistent with past effects over the 1992–2021 period. Trends in the prevalence of mental illness by age group over the period 1992–2021 are depicted in Supplementary Figures 10–12, with the age of peak prevalence of schizophrenia consistent with that derived from the APC model.

## Discussion

Over the past three decades, the global prevalence and incidence of mental disorders have shown an upward trend (1). This increase is not only associated with changes in population size and structure but also closely linked to factors such as heightened societal environmental pressures, shifts in lifestyle and behavioral patterns, and ecological and environmental influences (23, 24). The present study reflects differences in the prevalence trends of anxiety disorders, depression and schizophrenia by gender, year, SDI level and region, and uses APC models to reflect differences in the three dimensions of anxiety disorders, depression and schizophrenia by age, period and birth cohort among countries with different SDI levels. Differences in prevalence trends for anxiety disorders, depression and schizophrenia were highlighted. In addition, from a public health perspective, the rising prevalence of mental health disorders poses a major public health challenge, with multidimensional impacts: at the individual level, it may increase the risk of communicable and non-communicable diseases and lead to accidental and intentional injury (25); at the socio-economic level, it may lead to a decrease in labor productivity and a crowding out of healthcare resources (2); and at the demographic level, it may aggravate health inequalities, with life expectancy being 10 years shorter than normal for people with mental disorders, with life expectancy being 10 years shorter than normal. Life expectancy for people with mental disorders is 10–20 years shorter than for the general population (26). Therefore, the tertiary prevention of mental disorders needs to be emphasized, and the demand for mental health services is increasing (27).

The need for this study was necessitated due to the changes in mental illnesses in people after the global epidemic experienced after 2019, the results of this study showed that the increase in the prevalence of anxiety disorders, depression & schizophrenia observed over three decades is consistent with previous studies that documented rising mental health problems globally (1). The significantly higher prevalence and onset of anxiety and depression in females than males may include differences in the biological effects of puberty, and social factors during adolescence (28, 29), and a study has shown that females are more susceptible to anxiety disorders and depression at various stages of their reproductive lives, including puberty, menstruation, pregnancy, postpartum, and menopause (28). These periods are characterized by significant hormonal changes, suggesting a role for sex hormones in the onset, progression, and persistence of anxiety disorders and depression in women. It may also result from girls being more vulnerable to emotions (30). However, in schizophrenia, the opposite is true and is more severe in men. The present results are consistent with the McGrath J study (31). It may be related to the fact that adult women’s sex hormone levels fluctuate during the menstrual cycle, making the cyclical effects of high and low female hormones induce specific responses in the adult female brain, all of which have an effect on schizophrenia (32).

The global distribution of the prevalence of mental disorders in 2011–2021 shows that anxiety disorders continue to be more prevalent in North America, South America and Europe; depressive symptoms are more prevalent in Africa and Asia; and schizophrenia is more prevalent in North America, East Asia and Oceania. However, SDI levels vary across countries, as do the prevalence and incidence of different psychiatric disorders. The prevalence and incidence of anxiety disorders is higher in countries with high and medium levels of SDI, and countries with higher prevalence of anxiety disorders are concentrated in North and South America and European countries (33), which may be due to industrialization and urbanization over the past 40 years, as well as increased stress and competition at school, high parental expectations, and fast-changing socio-economic conditions, all of which have the potential to lead to an increase in the prevalence of anxiety disorders (34–36).

The higher prevalence of depression in countries in East Asia and Africa can be attributed to the compounding effects of surging social stress, structural imbalances in mental health services, and dramatic demographic changes during the economic transition (37, 38). From the economic perspective, these countries are in a stage of rapid industrialization and urbanization, but the uneven distribution of the dividends of growth has led to conflicts: East Asia is facing structural unemployment caused by automation in the manufacturing industry, while Africa is facing a reservoir of idle labor due to the expansion of the youth population and the shortage of employment opportunities; at the same time, the uncontrolled migration of the rural population to the cities has increased social segregation, housing overcrowding, and the lack of employment opportunities (37, 38). At the same time, uncontrolled rural–urban migration has exacerbated social segregation, overcrowded housing and environmental pollution, and weakened the psychological support function of traditional communities (39–41). Government resources are focused on the admission and treatment of psychiatric patients with violent tendencies or uncontrolled behaviors, while the mild-to-moderate population, especially the older adult, who make up the majority of depressed patients, have been marginalized for a long time (42). Factors such as chronic illness and emotional isolation in the context of population aging significantly increase the risk of depression in old age. This phenomenon is particularly prominent in East Asian and African countries, reflecting a structural tension between economic development and investment in mental health resources (43, 44).

The global prevalence of schizophrenia has not changed significantly over the past 30 years, but the prevalence of schizophrenia varies across countries with different SDI levels, the prevalence of schizophrenia is concentrated in the North American states as well as in East Asia and Oceania, which may be due to factors related to cultural diversity, with a high proportion of immigrant populations in the Australian or Canadian regions, which leads to individuals separated by cultural boundaries or conflict between social groups, studies have shown that first-generation immigrants have 2–3 times the incidence of schizophrenia than the native population due to language barriers, social isolation and discrimination, and that refugee populations have a higher risk of co-morbidity with schizophrenia due to post-traumatic stress disorder. This leads to a higher incidence of schizophrenia (45, 46). Students who face greater psychological stress due to social discrimination and bullying, and early childhood adversity, including bullying, may be at increased risk for schizophrenia through the mechanism of chronic stress-dopamine dysregulation (47); among other factors, birth and early life factors are important etiological factors in schizophrenia (48).

Joinpoint and age-period-cohort (APC) analyses demonstrate a rapid global increase in anxiety incidence across countries of varying Socio-demographic Index (SDI) levels post-2019, potentially driven by multiple synergistic mechanisms. Adolescents and young adults exhibited heightened vulnerability to mental health impacts from pandemic-related unemployment and educational disruptions, exacerbating psychological distress (49). Concurrently, overwhelmed healthcare systems resulted in delayed or interrupted mental health interventions, leaving pre-existing anxiety symptoms unmanaged (50). Direct psychosocial stressors, including COVID-19 health threats, mortality risks, and social isolation policies, further amplified population-level anxiety (51). The normalization of remote work and online education contributed to prolonged screen time and diminished face-to-face social interactions, compounding psychological strain (52). Additionally, reduced mental health stigma and improved public awareness likely increased symptom disclosure rates, partially inflating statistical measures of incidence (53). In terms of depression, the prevalence of depression was relatively stable, with a slight upward trend, except in countries with high SDI levels, where, interestingly, the prevalence of depression showed a decreasing trend between 1996 and 1999, which may be due to universal access to mental health services and early interventions (54), but a gradual increase after 2010, possibly due to the culture of social media, and economic instability that exacerbates the risk of morbidity, leading to accumulation of patients (55, 56).

The observed disparities in mental disorder trends across countries with varying Socio-demographic Index (SDI) levels may arise from the direct impacts of socioeconomic development: in high-SDI nations, well-established healthcare systems likely enhance the detection rates of mental disorders, yet aging populations and competitive societal pressures may concurrently elevate true prevalence rates (57); conversely, in low-SDI countries, structural stressors such as poverty, armed conflicts, and infectious diseases exacerbate mental health risks, though underdiagnosis persists due to limited healthcare access and cultural stigma (58). This divergence is further compounded by epidemiological transition patterns—high-SDI regions exhibit a higher burden of chronic mental disorders linked to lifestyle factors, whereas low-SDI settings remain dominated by infection-associated conditions and trauma-related disorders (59, 60). Additionally, methodological disparities in data quality and diagnostic validity contribute to cross-national discrepancies: high-SDI countries benefit from comprehensive health surveillance systems, while low-SDI regions often rely on fragmented surveys or modeled estimates. Critically, the limited cultural adaptability of Western-developed diagnostic tools in non-Western contexts may introduce measurement bias, undermining cross-country comparability (61).

### Limitation

Despite the application of data standardization and cleaning protocols within the Global Burden of Disease (GBD) framework (62–64), this study is subject to limitations inherent to the GBD 2021 dataset. The integration of heterogeneous data sources may introduce biases and uncertainties in the estimates, particularly for regions with sparse primary data. The reliance on modeled estimates for areas lacking direct epidemiological measurements—common in low-resource settings—raises concerns about validity, especially in Africa, where under-resourced healthcare systems and the underprioritization of mental health research and treatment likely contribute to systematic underestimation of true disease incidence. While age-standardization methods were employed to mitigate potential underestimation biases, residual inaccuracies may persist due to incomplete diagnostic coverage and cultural barriers to reporting mental disorders. These limitations necessitate cautious interpretation of cross-regional comparisons and underscore the imperative for localized validation studies.

## Conclusion

This study analyses three decades of global burden of disease data to systematically reveal the evolution of mental health problems in response to societal development, highlighting the complex interplay of age, period and cohort influences on the impact of anxiety disorders, depression and schizophrenia. Different SDI levels all exhibited unique trends reflecting their specific socioeconomic, cultural, and historical contexts, suggesting that tailored public health strategies are essential to effectively address and manage the prevalence of anxiety disorders, depression, and schizophrenia in these different settings. Future research should examine in greater depth the impact of specific policies and interventions to ensure an integrated approach to the management of anxiety, depression and schizophrenia in these rapidly developing countries.

## Data Availability

Publicly available datasets were analyzed in this study. This data can be found here: https://vizhub.healthdata.org/gbd-results.
