# Supplementary material for: GBD: incidence rates and prevalence of anxiety disorders, depression and schizophrenia in countries with different SDI levels, 1990–2021
Source: Front Public Health. 2025 May 16;13:1556981. doi: 10.3389/fpubh.2025.1556981 (PMC12124139; doi:10.3389/fpubh.2025.1556981)
Supplement: Supplementary file 1 [file Data_Sheet_1.pdf]

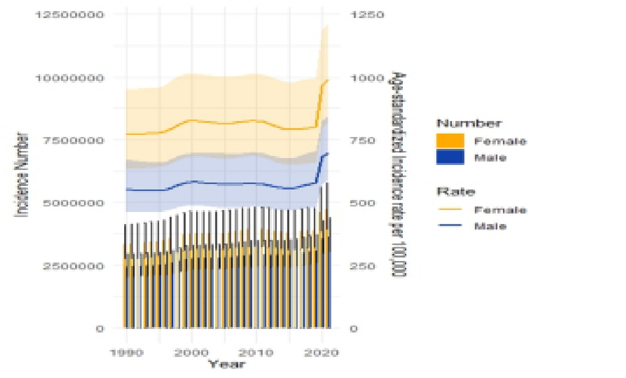

Incidence of Anxiety disorders in High SDI, 1990 to 2021

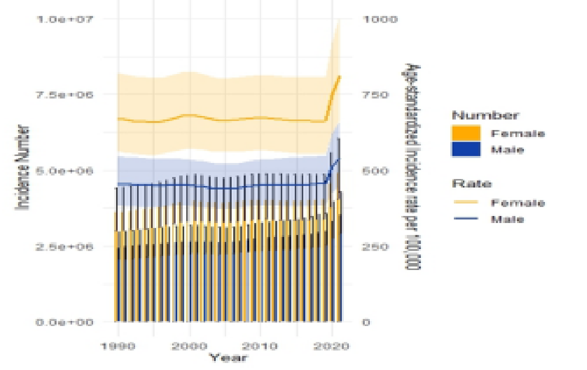

Incidence of Anxiety disorders in High-middle SDI, 1990 to 2021

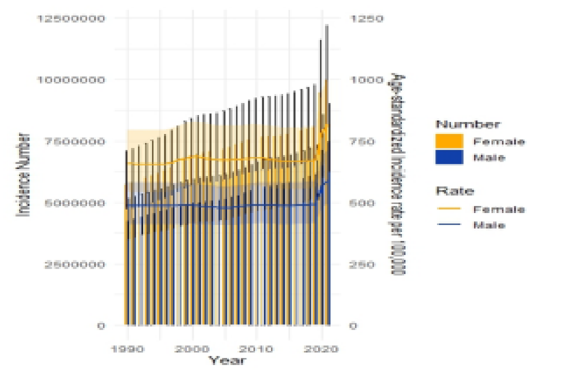

Incidence of Anxiety disorders in Middle SDI, 1990 to 2021

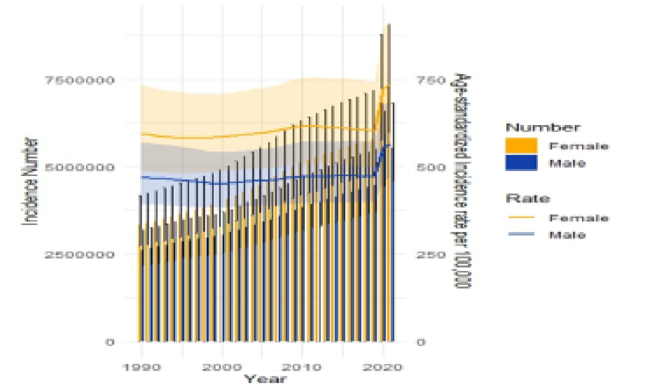

Incidence of Anxiety disorders in Low-middle SDI, 1990 to 2021

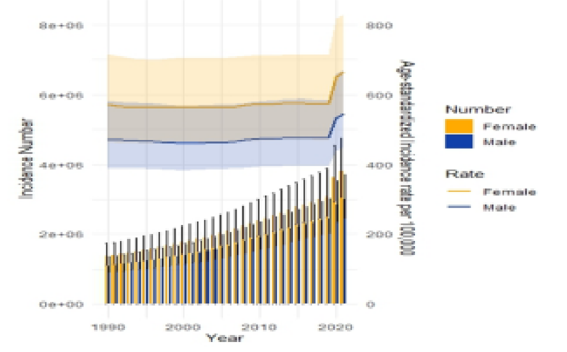

Incidence of Anxiety disorders in Low SDI, 1990 to 2021

Supplementary figure 1: Incidence of Anxiety disorders at different SDI levels, 1990 to 2021

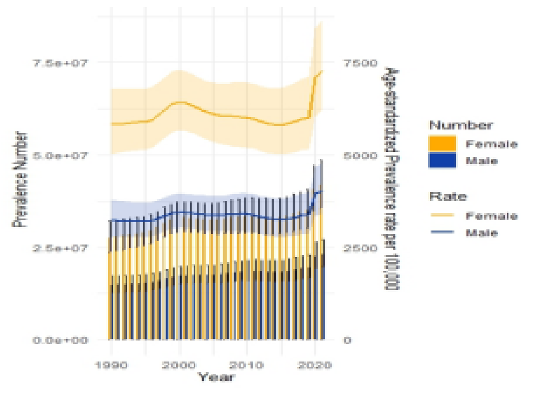

Prevalence of Anxiety disordersin High SDI, 1990 to 2021

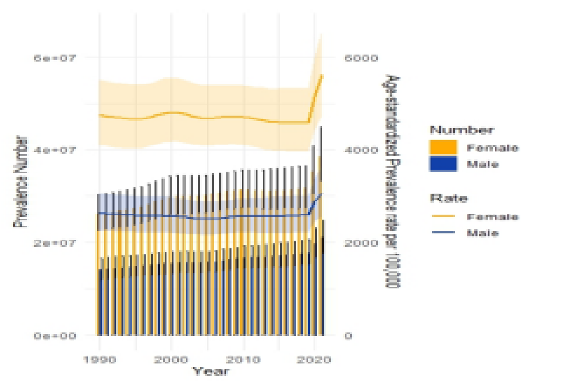

Prevalence of Anxiety disordersin High-middle SDI, 1990 to 2021

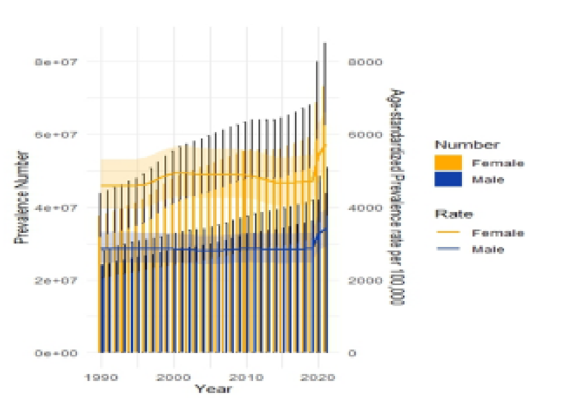

Prevalence of Anxiety disordersin Middle SDI, 1990 to 2021

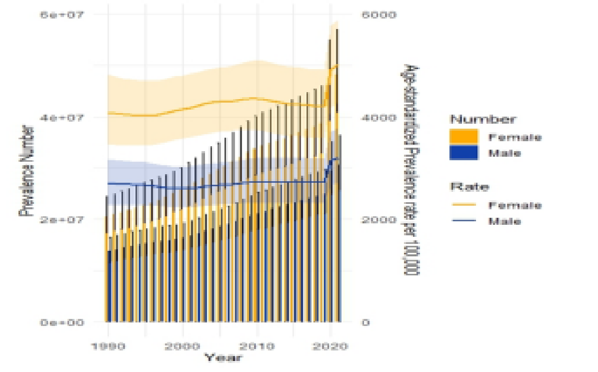

Prevalence of Anxiety disordersin Low-middle SDI, 1990 to 2021

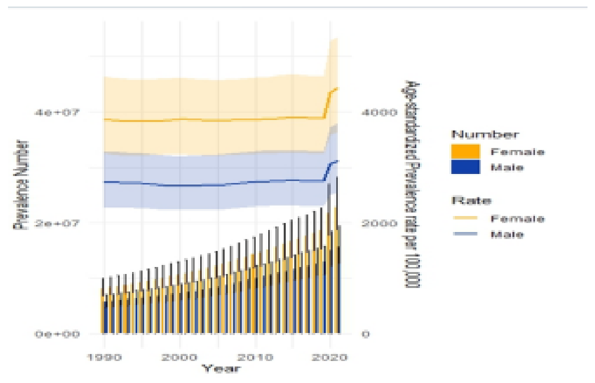

Prevalence of Anxiety disordersin Low SDI, 1990 to 2021

Supplementary figure 2: Prevalence of Anxiety disorders at different SDI levels, 1990 to 2021

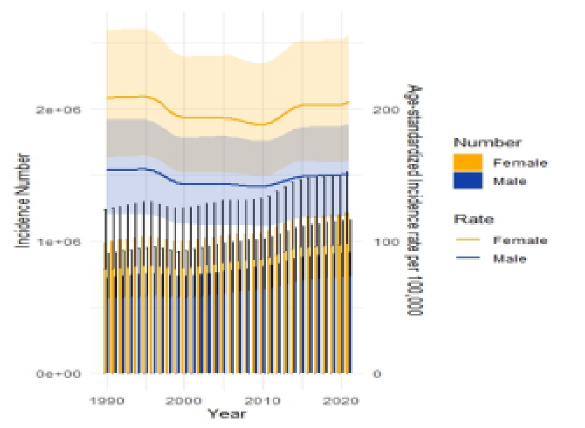

Incidence of Dysthymia in High SDI, 1990 to 2021

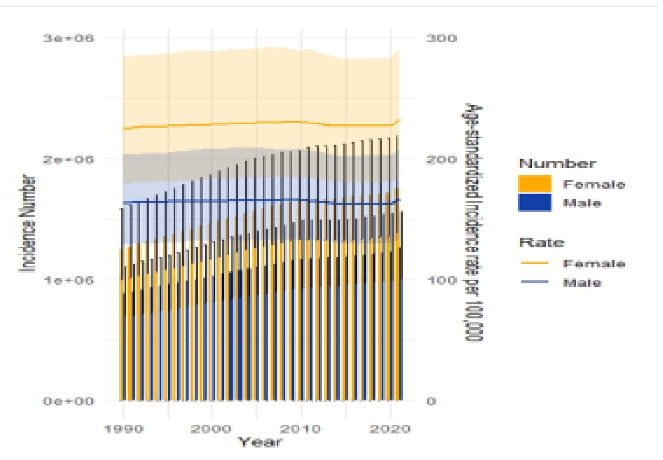

Incidence of Dysthymia in High-middle SDI, 1990 to 2021

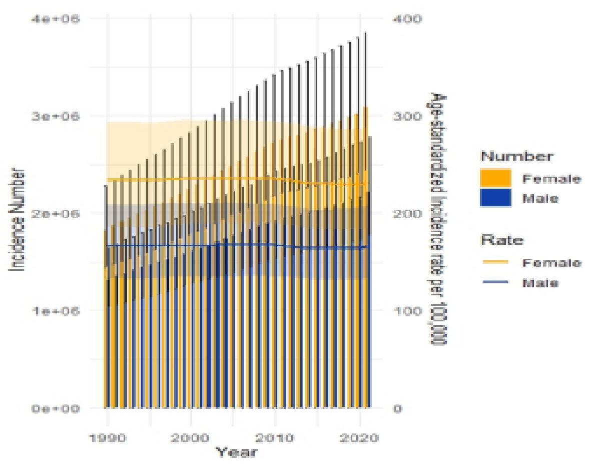

Incidence of Dysthymia in Middle SDI, 1990 to 2021

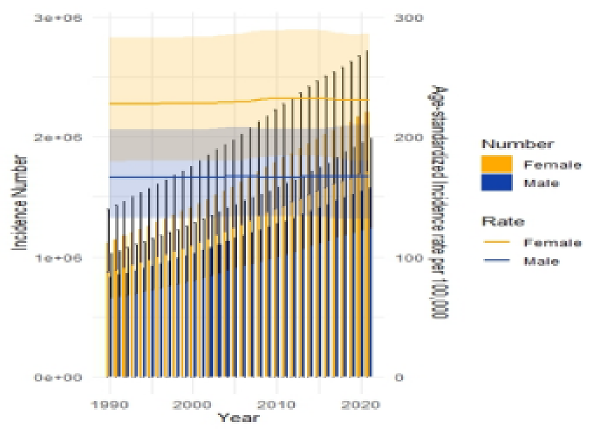

Incidence of Dysthymia in Low-middle SDI, 1990 to 2021

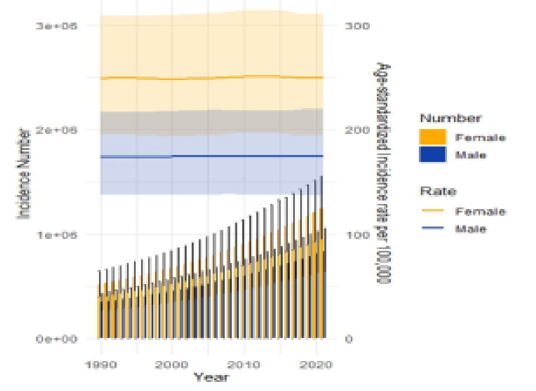

Incidence of Dysthymia in Low SDI, 1990 to 2021

Supplementary figure 3: Incidence of Depression at different SDI levels, 1990 to 2021

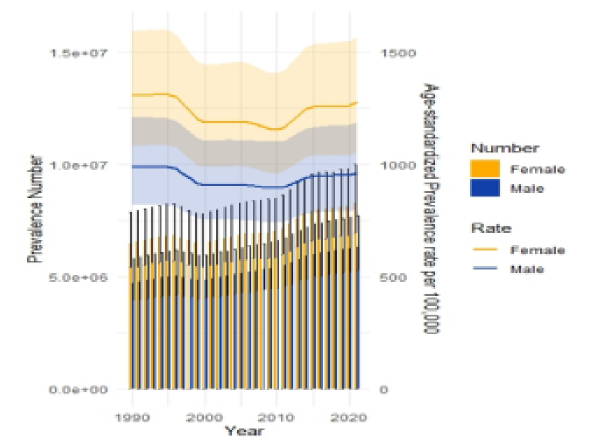

Prevalence of Dysthymia in High SDI, 1990 to 2021

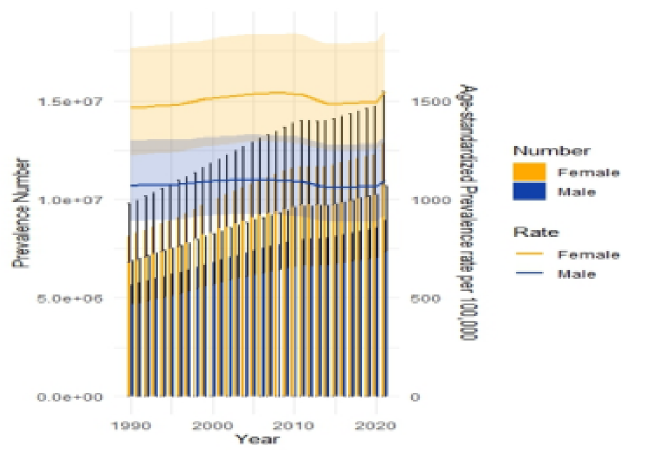

Prevalence of Dysthymia in High-middle SDI, 1990 to 2021

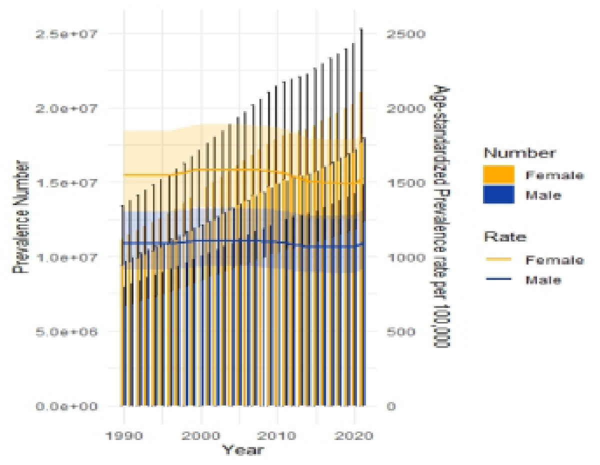

Prevalence of Dysthymia in Middle SDI, 1990 to 2021

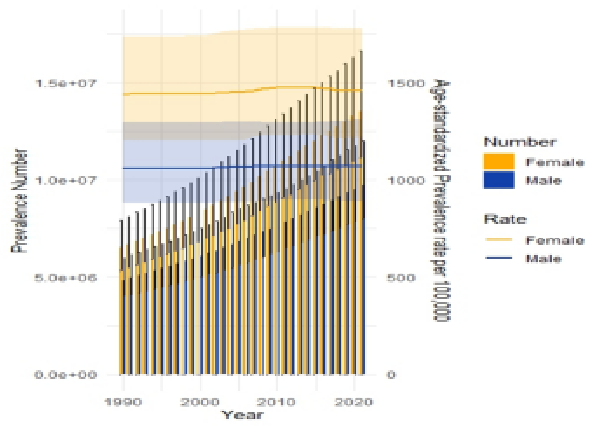

Prevalence of Dysthymia in Low-middle SDI, 1990 to 2021

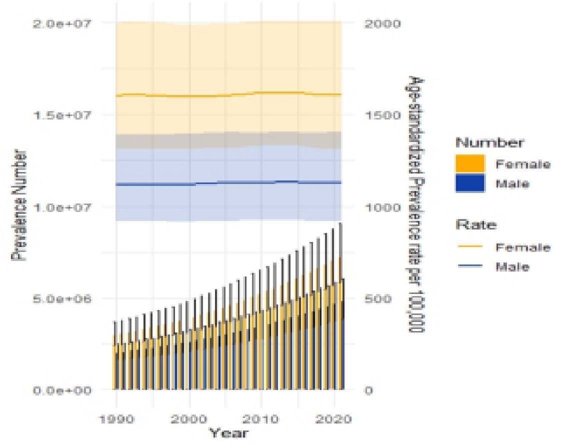

Prevalence of Dysthymia in Low SDI, 1990 to 2021

Supplementary figure 4: Prevalence of Depression at different SDI levels, 1990 to 2021

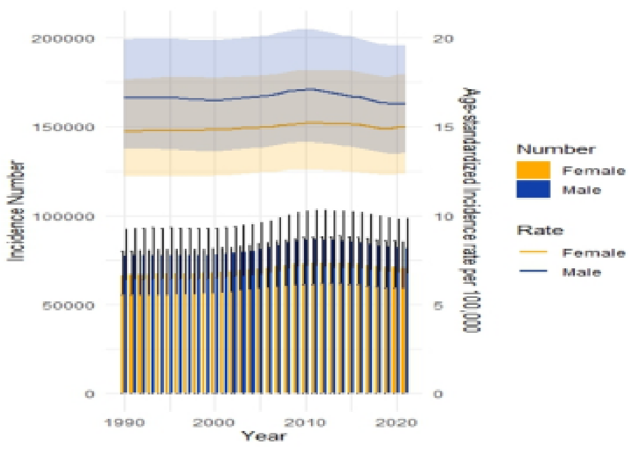

Incidence of Schizophrenia in High SDI, 1990 to 2021

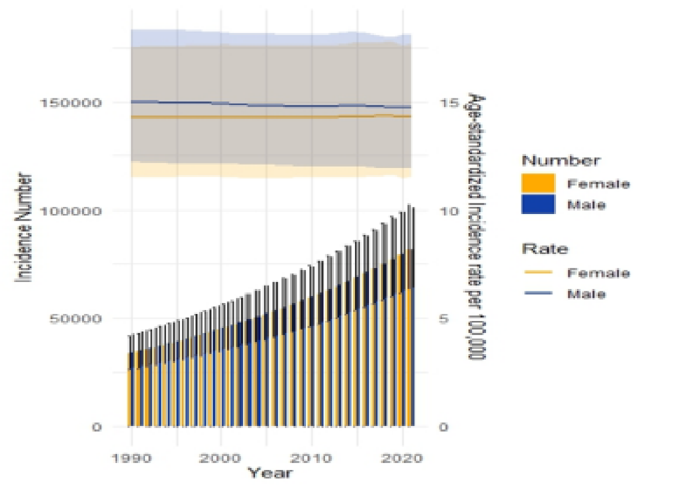

Incidence of Schizophrenia in High-middle SDI, 1990 to 2021

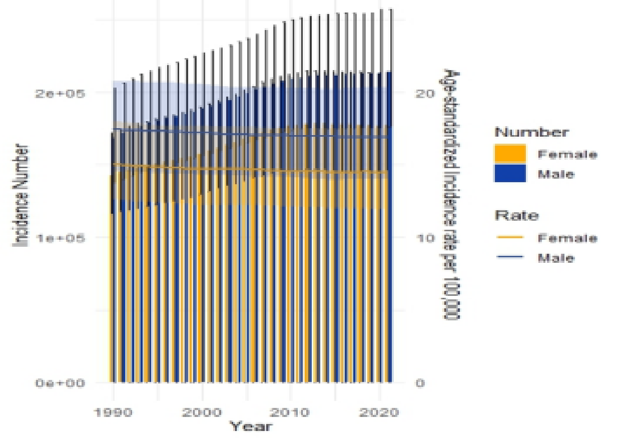

Incidence of Schizophrenia in Middle SDI, 1990 to 2021

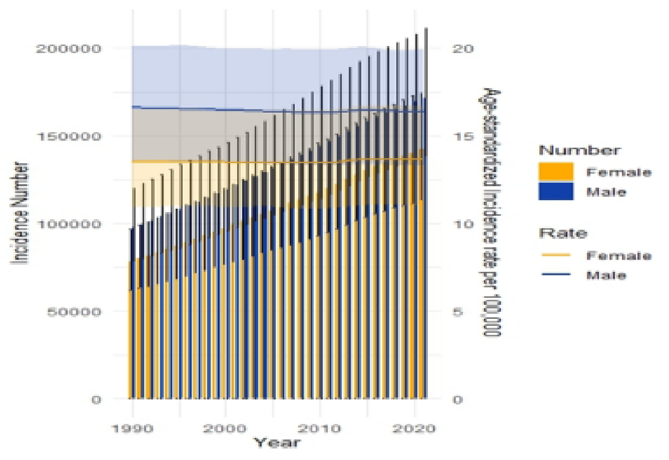

Incidence of Schizophrenia in Low-middle SDI, 1990 to 2021

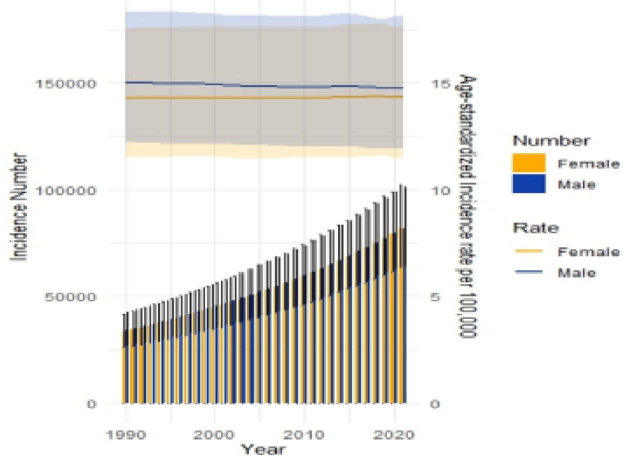

Incidence of Schizophrenia in Low SDI, 1990 to 2021

Supplementary figure 5: Incidence of Schizophrenia at different SDI levels, 1990 to 2021

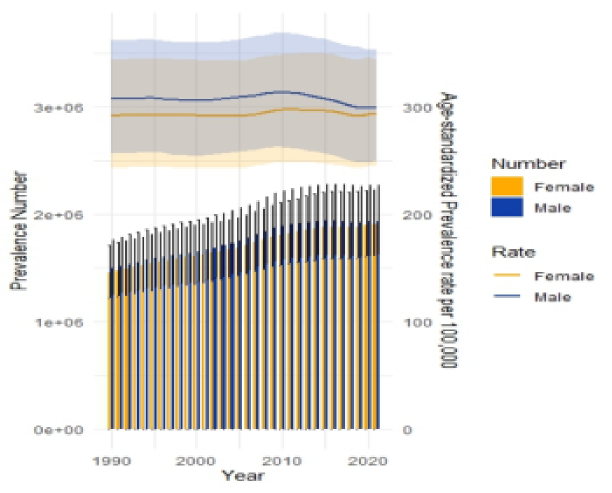

Prevalence of Schizophrenia in High SDI, 1990 to 2021

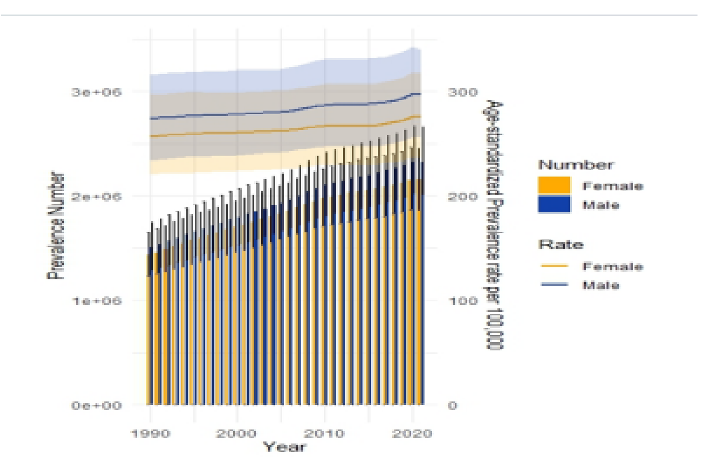

Prevalence of Schizophrenia in High-middle SDI, 1990 to 2021

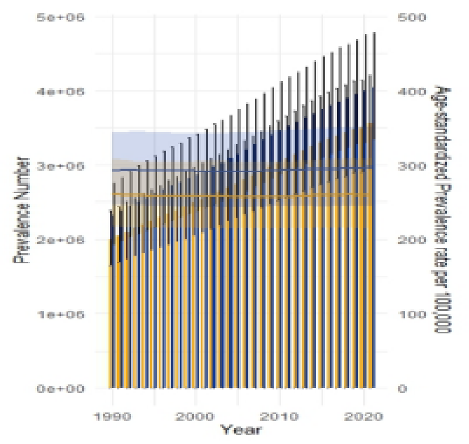

Prevalence of Schizophrenia in Middle SDI, 1990 to 2021

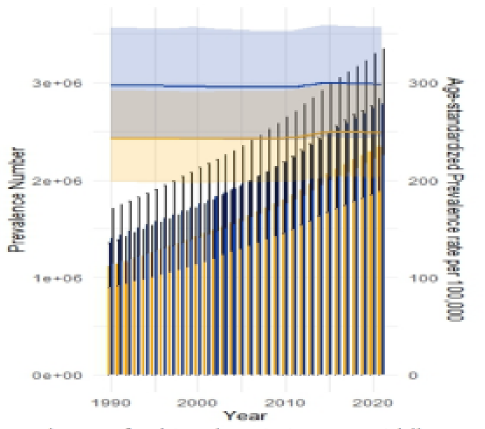

Prevalence of Schizophrenia in Low-middle SDI, 1990 to 2021

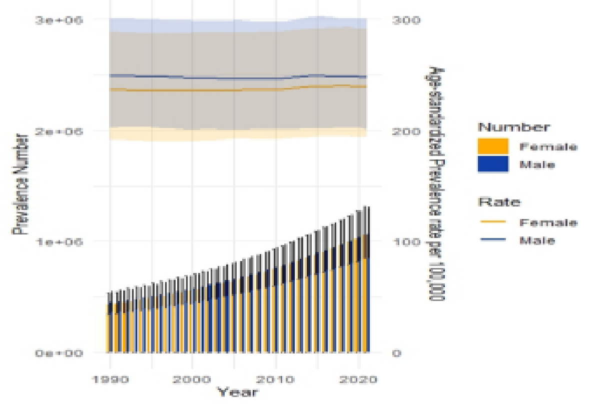

Prevalence of Schizophrenia in Low SDI, 1990 to 2021

Supplementary figure 6: Prevalence of Schizophrenia at different SDI levels, 1990 to 2021

Supplementary Table 1: Incidence of Anxiety disorders at Different SDI Levels from 1990 to 2021joinponit Calculated by AAPC			
different SDI levels	Incidence_AAPC		
	Both	Female	Male
High SDI	0.87 (0.61 - 1.14)	0.97 (0.76 - 1.18)	0.86 (0.60 - 1.11)
High-middle SDI	0.62 (0.56 - 0.68)	0.65 (0.59 - 0.71)	0.61 (0.46 - 0.76)
Middle SDI	0.74 (0.58 - 0.89)	0.83 (0.67 - 0.99)	0.72 (0.58 - 0.85)
Low-middle SDI	0.74 (0.58 - 0.90)	0.86 (0.63 - 1.09)	0.04 (0.04 - 0.05)
Low SDI	0.55 (0.49 - 0.61)	0.61 (0.51 - 0.71)	0.52 (0.45 - 0.58)

Supplementary Table2: Incidence of Depression at Different SDI Levels from 1990 to 2021joinponit Calculated by AAPC	
different SDI	Incidence_AAPC

levels	Both	Female	Male
High SDI	-0.08 (-0.15 - -0.02)	-0.06(-0.08--0.03)	-0.07(-0.16 - 0.01)
High-middle SDI	0.05 (0.04 - 0.07)	0.08(0.07 - 0.10)	0.04(0.03 - 0.05)
Middle SDI	-0.01 (-0.04 - 0.01)	-0.03(-0.06 - 0.01)	-0.01(-0.02 - 0.00)
Low-middle SDI	0.04 (0.04 - 0.05)	0.04 (0.04 - 0.05)	0.02 (0.02 - 0.02)
Low SDI	0.01 (0.01 - 0.01)	0.00 (-0.00 - 0.01)	0.01 (0.01 - 0.01)

Supplementary Table3: Incidence of Schizophrenia at Different SDI Levels from 1990 to 2021 joinponit Calculated by AAPC			
different SDI levels	Incidence_AAPC		
	Both	Female	Male
High SDI	-0.00 (-0.01 - 0.01)	0.06 (0.05- 0.06)	-0.05(-0.08--0.02)
High-middle SDI	0.16 (0.13 - 0.18)	0.13 (0.11- 0.14)	0.17 (0.14 - 0.20)
Middle SDI	-0.11(-0.11 - -0.10)	-0.12(-0.14--0.11)	-0.09(-0.10--0.08)
Low-middle SDI	-0.01 (-0.01 - -0.01)	0.04 (0.03 - 0.04)	-0.04(-0.05--0.04)
Low SDI	-0.02 (-0.03 - -0.01)	0.01 (0.00 - 0.02)	-0.05(-0.05--0.04)

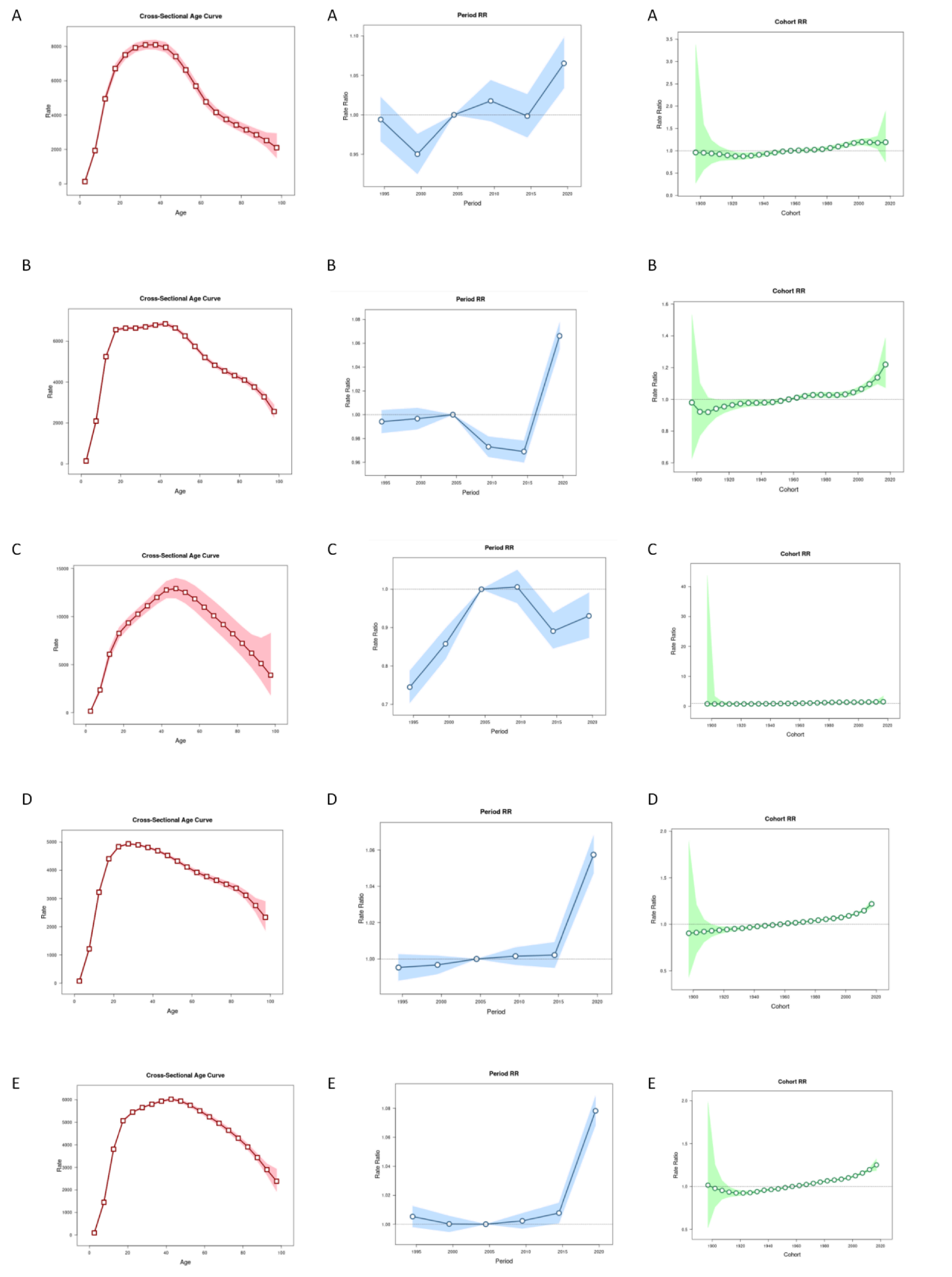

Supplementary figure 7 : Age- period- cohort effects on Anxiety disorders prevalence rates from 1992 to 2021.(A)Australia ;(B)Argentina;(C)Brazil;(D)Angola ;(E)Haiti.

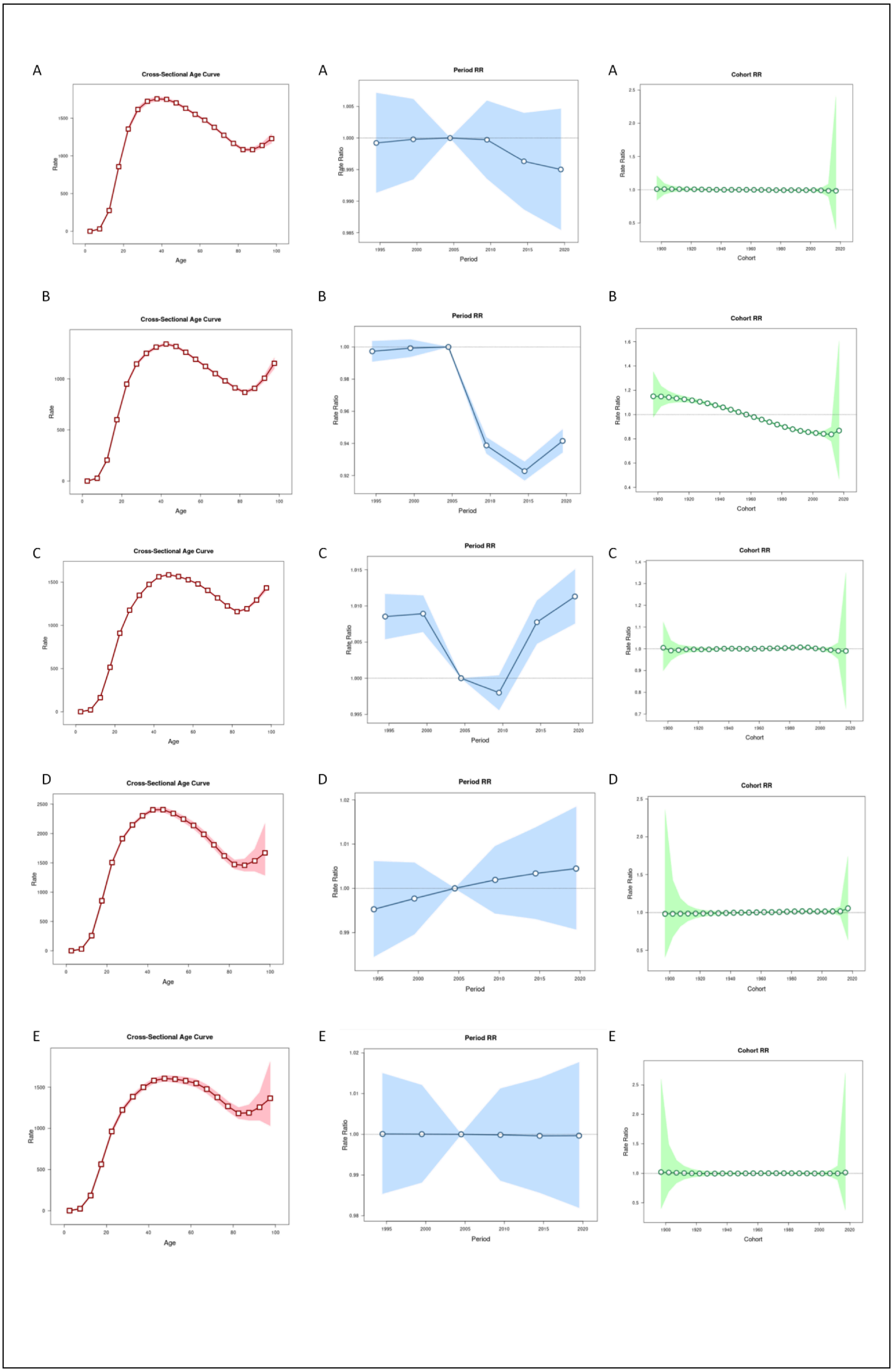
A
Cross-Sectional Age Curve
Rate
Age
A
Period RR
Rate Ratio
Period
A
Cohort RR
Rate Ratio
Cohort
B
Cross-Sectional Age Curve
Rate
Age
B
Period RR
Rate Ratio
Period
B
Cohort RR
Rate Ratio
Cohort
C
Cross-Sectional Age Curve
Rate
Age
C
Period RR
Rate Ratio
Period
C
Cohort RR
Rate Ratio
Cohort
D
Cross-Sectional Age Curve
Rate
Age
D
Period RR
Rate Ratio
Period
D
Cohort RR
Rate Ratio
Cohort
E
Cross-Sectional Age Curve
Rate
Age
E
Period RR
Rate Ratio
Period
E
Cohort RR
Rate Ratio
Cohort

Supplementary figure 8 : Age- period- cohort effects on Depression prevalence rates from 1992 to 2021.(A)Australia ;(B)Argentina;(C)Brazil;(D)Angola ;(E)Haiti.

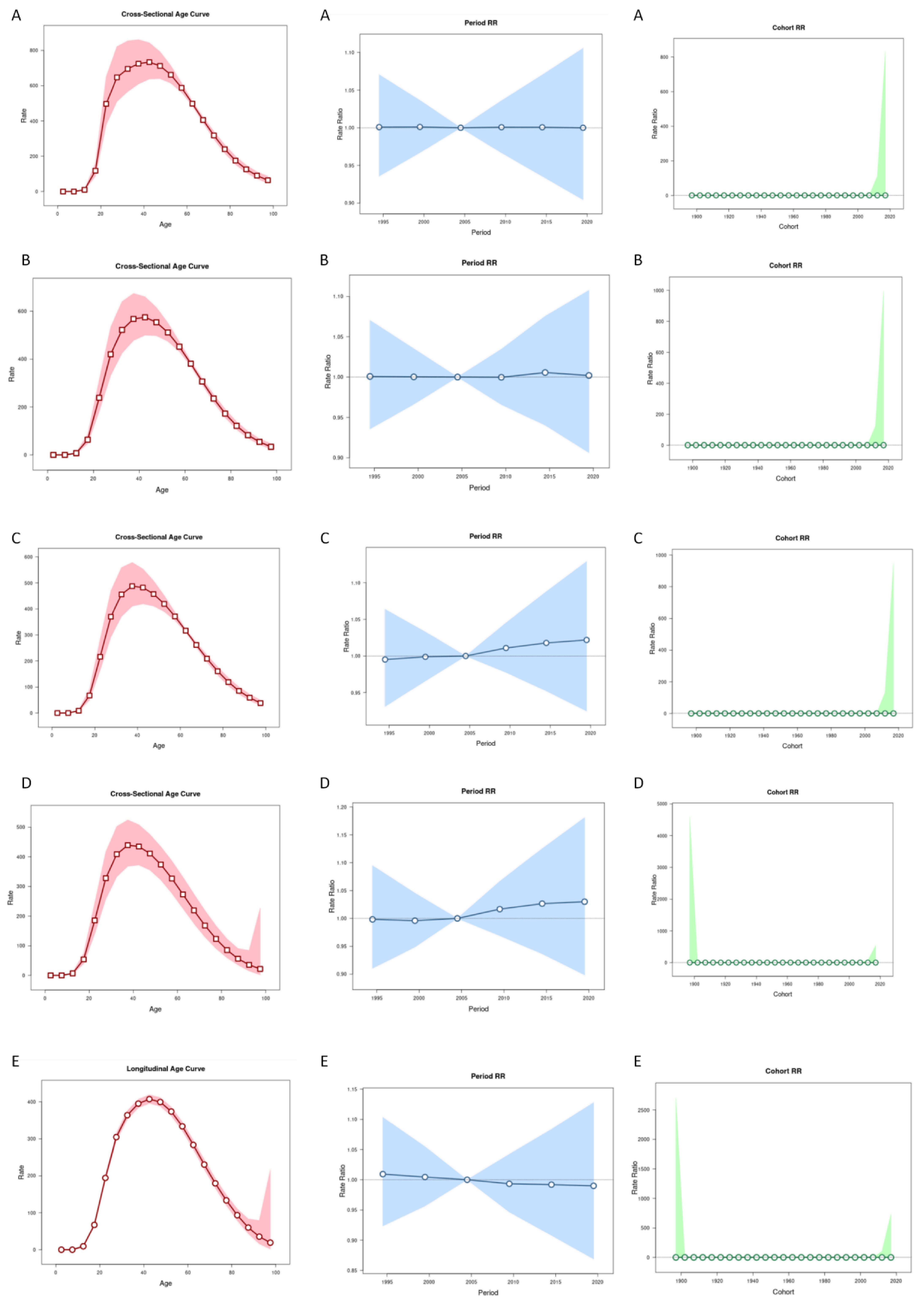

Supplementary figure 9 : Age- period- cohort effects on Schizophrenia prevalence rates from 1992 to

2021.(A)Australia ;(B)Argentina;(C)Brazil;(D)Angola ;(E)Haiti.

Supplementary Table 4: APC (Age-Period-Cohort) Model Analysis of Anxiety Disorder Prevalence in Countries Across Different SDI Levels, 1992- 2021			
	Net Drift (% /year)	CI.Lo	CI.Hi
Australia	0.293	0.152	0.434
Argentina	0.136	0.087	0.185
Brazil	0.707	0.352	1.063
Angola	0.183	0.122	0.244
Haiti	0.215	0.159	0.27

Supplementary Table 5: APC (Age-Period-Cohort) Model Analysis of Depression Prevalence in Countries Across Different SDI Levels, 1992-2021			
	Net Drift (% /year)	CI.Lo	CI.Hi
Australia	-0.018	-0.074	0.038
Argentina	-0.336	-0.378	-0.295
Brazil	0.005	-0.017	0.026
Angola	0.037	-0.047	0.121
Haiti	-0.002	-0.105	0.102

Supplementary Table 6: APC (Age-Period-Cohort) Model Analysis of Schizophrenia Prevalence in Countries Across Different SDI Levels, 1992-2021			
	Net Drift (% /year)	CILo	CIHi
Australia	-0.003	-0.667	0.666
Argentina	0.012	-0.652	0.681
Brazil	0.114	-0.549	0.781
Angola	0.151	-0.751	1.062
Haiti	-0.081	-0.936	0.781

**Supplementary Table 7. Wald Tests for the Age-Period-Cohort Model Analysis of Anxiety Disorder Prevalence Across Countries Stratified by SDI Level (1992- 2021)**				
		x2	df	p
Australia	NetDrift=0	16.704	1	<0.001
	All Age Deviations=0	4541.6957	18	<0.001
	All Period Deviations=0	38.1892	4	<0.001
	All Cohort Deviations=0	17.2937	23	0.7946
	All Period RR=1	55.5687	5	<0.001
	All Cohort RR=1	90.9802	24	<0.001
	All Local Drifts=Net Drift	17.1635	20	0.6423
Argentina	NetDrift=0	29.6625	1	<0.001
	All Age Deviations=0	37175.5455	18	<0.001
	All Period Deviations=0	592.9527	4	<0.001
	All Cohort Deviations=0	53.7081	23	0.0003
	All Period RR=1	628.0631	5	<0.001

	All Cohort RR = 1	132.7142	24	<0.001
	All Local Drifts = Net Drift	53.7002	20	0.0001
Brazil	NetDrift = 0	15.2905	1	0.0001
	All Age Deviations = 0	1327.9292	18	<0.001
	All Period Deviations = 0	170.1625	4	<0.001
	All Cohort Deviations = 0	15.6865	23	0.8684
	All Period RR = 1	181.804	5	<0.001
	All Cohort RR = 1	115.8654	24	<0.001
	All Local Drifts = Net Drift	15.6107	20	0.7405
Angola	NetDrift = 0	34.725	1	<0.001
	All Age Deviations = 0	347418.3579	18	<0.001
	All Period Deviations = 0	906.3125	4	<0.001
	All Cohort Deviations = 0	103.2938	23	<0.001
	All Period RR = 1	946.2836	5	<0.001
	All Cohort RR = 1	1041.097	24	<0.001
	All Local Drifts = Net Drift	99.362	20	<0.001
Haiti	NetDrift = 0	57.206	1	<0.001
	All Age Deviations = 0	167460.5012	18	<0.001
	All Period Deviations = 0	1046.3958	4	<0.001
	All Cohort Deviations = 0	72.7992	23	<0.001
	All Period RR = 1	1109.3394	5	<0.001
	All Cohort RR = 1	1173.7018	24	<0.001
	All Local Drifts = Net Drift	72.3277	20	<0.001

Supplementary Table 8. Wald Tests for the Age-Period-Cohort Model Analysis of Depression Prevalence Across Countries Stratified by SDI Level (1992– 2021)				
		x2	df	p
Australia	NetDrift=0	0.4035	1	0.5253
	All Age Deviations=0	86812.2916	18	<0.001
	All Period Deviations=0	2.033	4	0.7297
	All Cohort Deviations=0	1.7001	23	1
	All Period RR=1	2.4825	5	0.7791
	All Cohort RR=1	4.4099	24	1
	All Local Drifts=Net Drift	1.6927	20	1
Argentina	NetDrift=0	253.6609	1	<0.001
	All Age Deviations=0	138303.0609	18	<0.001
	All Period Deviations=0	630.4767	4	<0.001
	All Cohort Deviations=0	58.1839	23	0.0001
	All Period RR=1	871.2092	5	<0.001
	All Cohort RR=1	2073.3134	24	<0.001
	All Local Drifts=Net Drift	57.9719	20	<0.001
Brazil	NetDrift=0	0.1853	1	0.6669
	All Age Deviations=0	714817.7359	18	<0.001
	All Period Deviations=0	235.1728	4	<0.001
	All Cohort Deviations=0	22.7426	23	0.4759
	All Period RR=1	236.0278	5	<0.001
	All Cohort RR=1	30.1871	24	0.1786

	All Local Drifts = Net Drift	21.5623	20	0.3647
Angola	NetDrift=0	0.745	1	0.3881
	All Age Deviations = 0	121685.3737	18	<0.001
	All Period Deviations = 0	0.2202	4	0.9944
	All Cohort Deviations = 0	3.8798	23	1
	All Period RR = 1	0.9425	5	0.9671
	All Cohort RR = 1	14.6462	24	0.9307
	All Local Drifts = Net Drift	3.5991	20	1
Haiti	NetDrift=0	0.0014	1	0.97
	All Age Deviations = 0	38955.8377	18	<0.001
	All Period Deviations = 0	0.0017	4	1
	All Cohort Deviations = 0	0.701	23	1
	All Period RR = 1	0.0031	5	1
	All Cohort RR = 1	0.7012	24	1
	All Local Drifts = Net Drift	0.6436	20	1

Supplementary Table 9. Wald Tests for the Age-Period-Cohort Model Analysis of Schizophrenia Prevalence Across Countries Stratified by SDI Level (1992– 2021)

		x2	df	p
Australia	NetDrift=0	0.0001	1	0.9932
	All Age Deviations = 0	44724.3758	18	<0.001
	All Period Deviations = 0	0.0601	4	0.9996
	All Cohort Deviations = 0	0.4153	23	1

	All Period RR = 1	0.0602	5	1
	All Cohort RR = 1	0.6346	24	1
	All Local Drifts = Net Drift	0.376	20	1
Argentina	NetDrift = 0	0.0013	1	0.971
	All Age Deviations = 0	79311.068	18	<0.001
	All Period Deviations = 0	2.1146	4	0.7147
	All Cohort Deviations = 0	1.7775	23	1
	All Period RR = 1	2.1158	5	0.8329
	All Cohort RR = 1	2.0373	24	1
	All Local Drifts = Net Drift	1.7762	20	1
Brazil	NetDrift = 0	0.1127	1	0.7371
	All Age Deviations = 0	329688.8378	18	<0.001
	All Period Deviations = 0	12.2808	4	0.0154
	All Cohort Deviations = 0	36.2315	23	0.0391
	All Period RR = 1	12.3935	5	0.0298
	All Cohort RR = 1	59.0032	24	0.0001
	All Local Drifts = Net Drift	36.1703	20	0.0147
Angola	NetDrift = 0	0.107	1	0.7436
	All Age Deviations = 0	25589.0998	18	<0.001
	All Period Deviations = 0	3.0499	4	0.5495
	All Cohort Deviations = 0	2.5854	23	1
	All Period RR = 1	3.1536	5	0.6763
	All Cohort RR = 1	18.6349	24	0.7712

	All Local Drifts = Net Drift	2.5721	20	1
Haiti	NetDrift = 0	0.0344	1	0.8529
	All Age Deviations = 0	11675.6971	18	<0.001
	All Period Deviations = 0	0.2172	4	0.9945
	All Cohort Deviations = 0	0.2171	23	1
	All Period RR = 1	0.2506	5	0.9985
	All Cohort RR = 1	2.5874	24	1
	All Local Drifts = Net Drift	0.2004	20	1

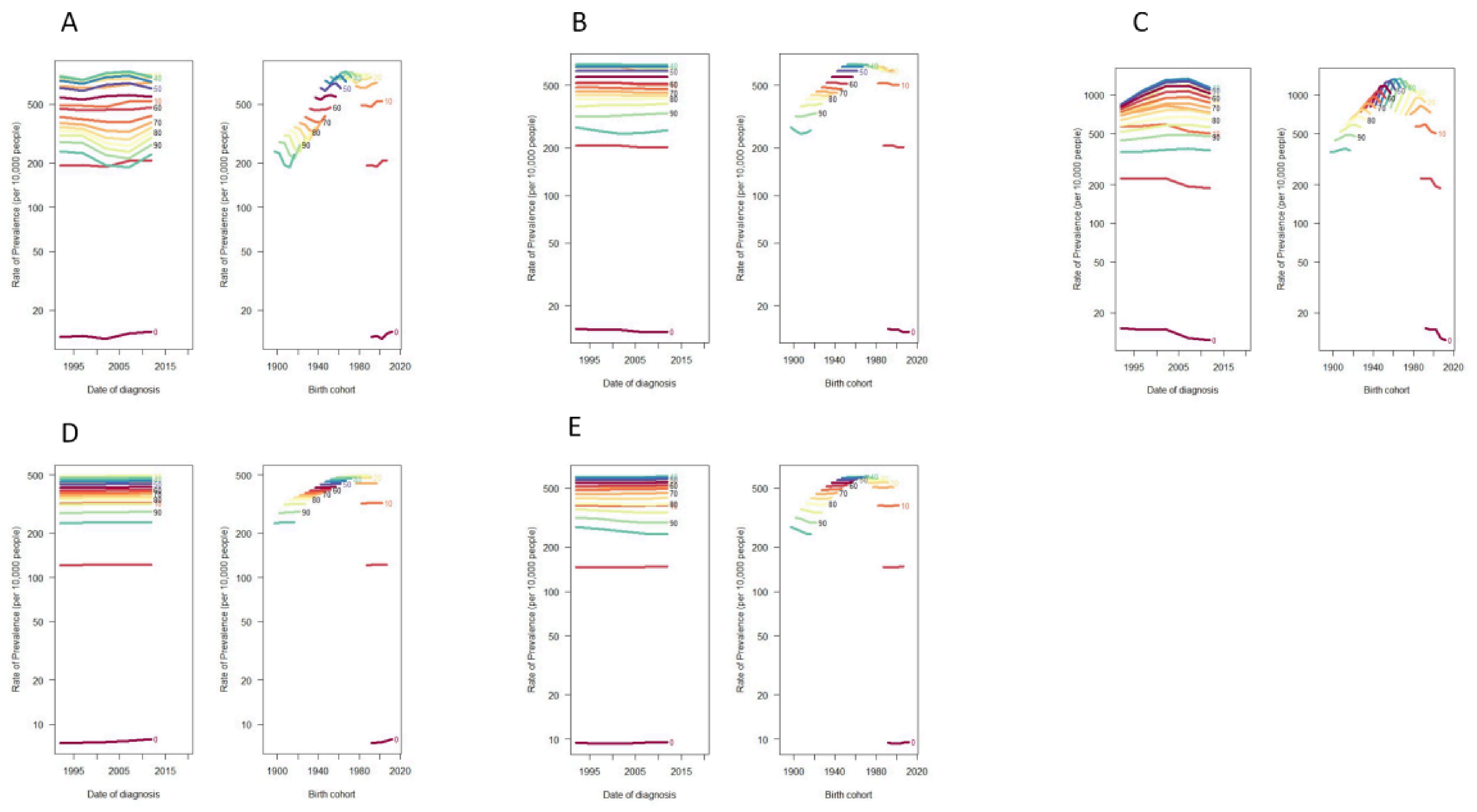

Supplementary Figure 10: Prevalence of Anxiety disorders in different SDI countries.(A)Australia ;(B)Argentina;(C)Brazil;(D)Angola ;(E)Haiti.

Left panel: period-specific prevalence by age group; each line connects birth cohort-specific prevalence in the 5-year age group; Right panel: birth cohort-specific prevalence by age group; each line connects birth cohort-specific prevalence in the 5-year age group.

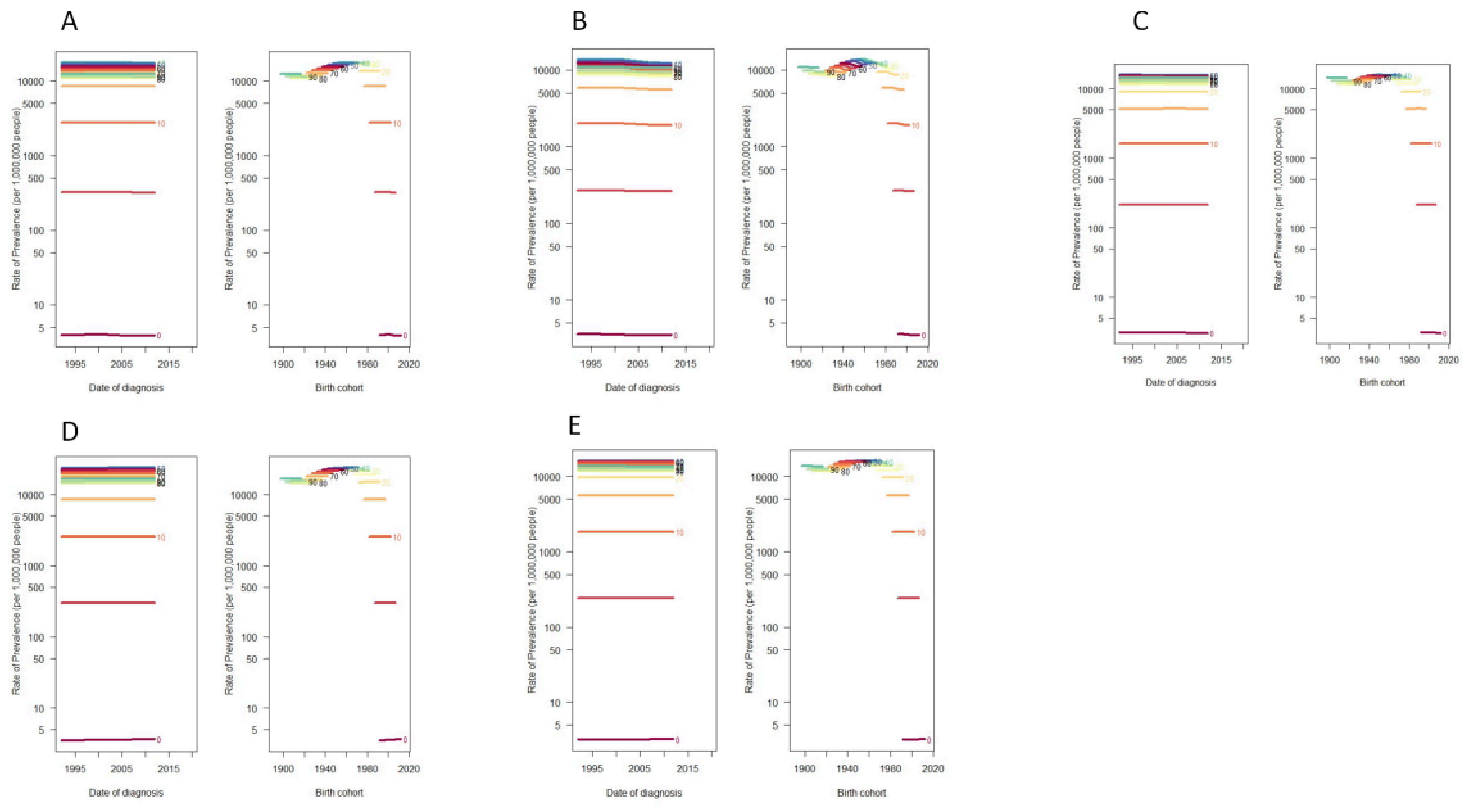

Supplementary Figure 11: Prevalence of Depression in different SDI countries.(A)Australia ;(B)Argentina;(C)Brazil;(D)Angola ;(E)Haiti.

Left panel: period-specific prevalence by age group; each line connects birth cohort-specific prevalence in the 5-year age group; Right panel: birth cohort-specific prevalence by age group; each line connects birth cohort-specific prevalence in the 5-year age group.

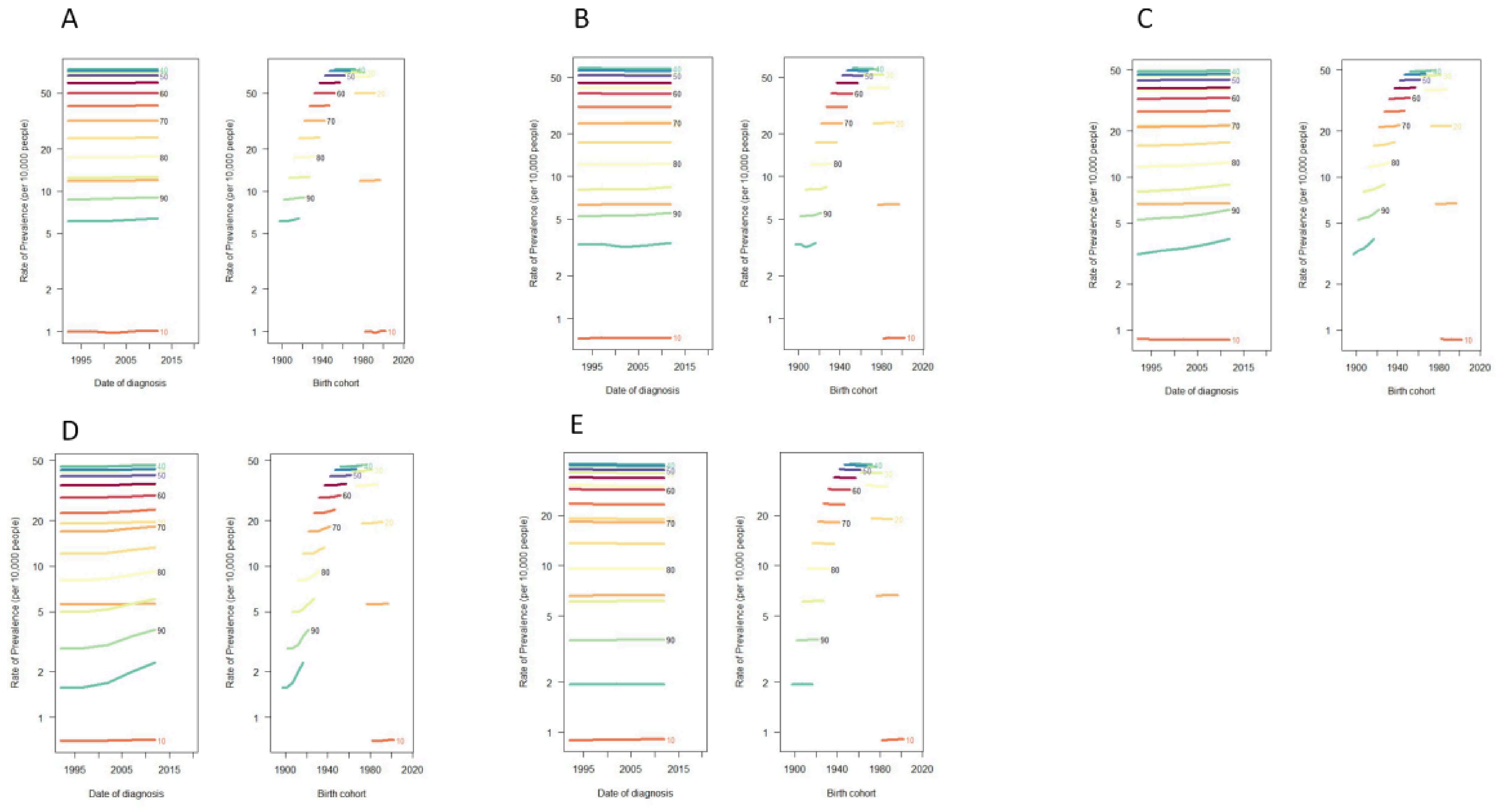

Supplementary Figure 12: Prevalence of Schizophrenia in different SDI countries.(A)Australia ;(B)Argentina;(C)Brazil;(D)Angola ;(E)Haiti.

Left panel: period-specific prevalence by age group; each line connects birth cohort-specific prevalence in the 5-year age group; Right panel: birth cohort-specific prevalence by age group; each line connects birth cohort-specific prevalence in the 5-year age group.

A

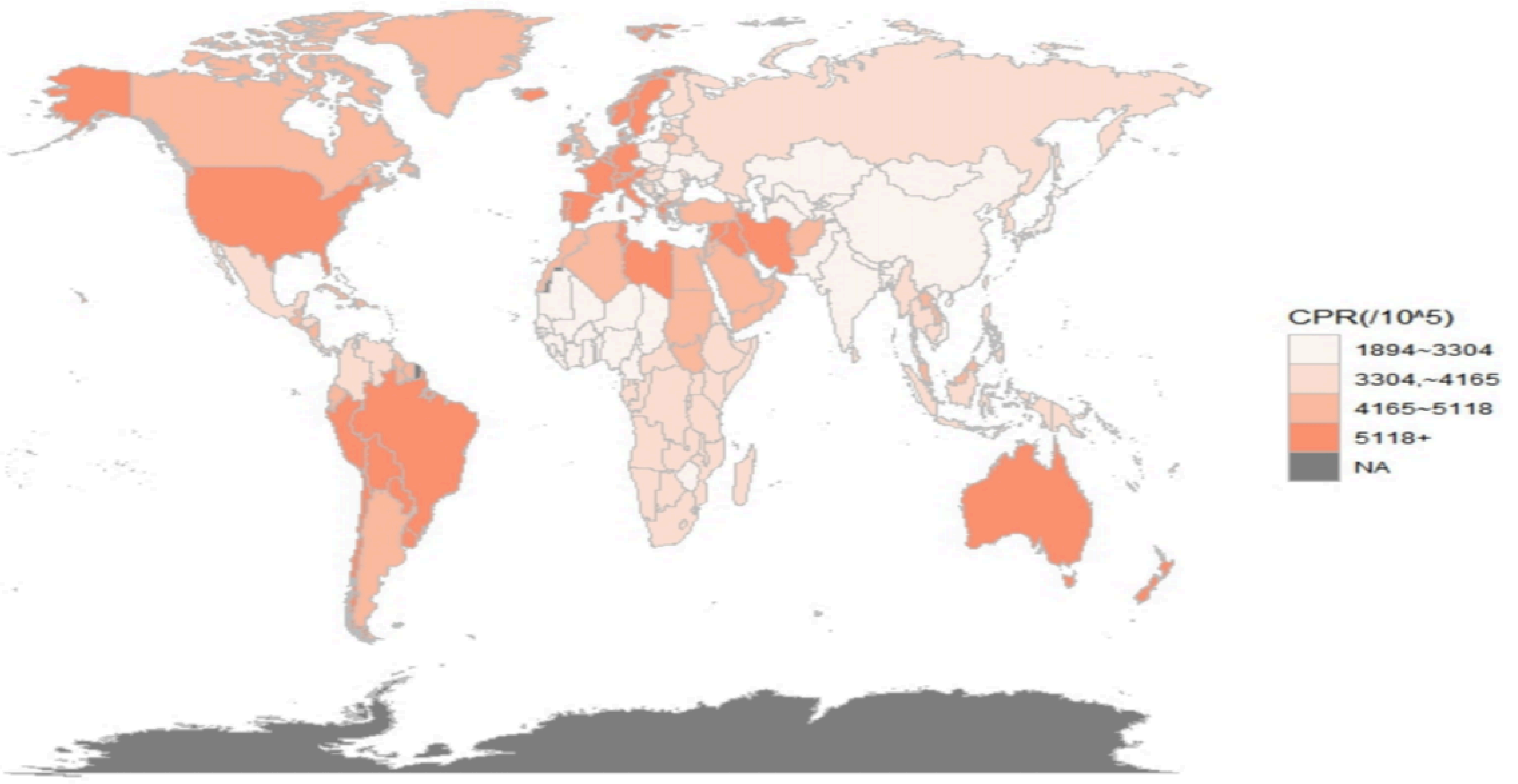
CPR(/10^5)
1894~3304
3304,~4165
4165~5118
5118+
NA

B

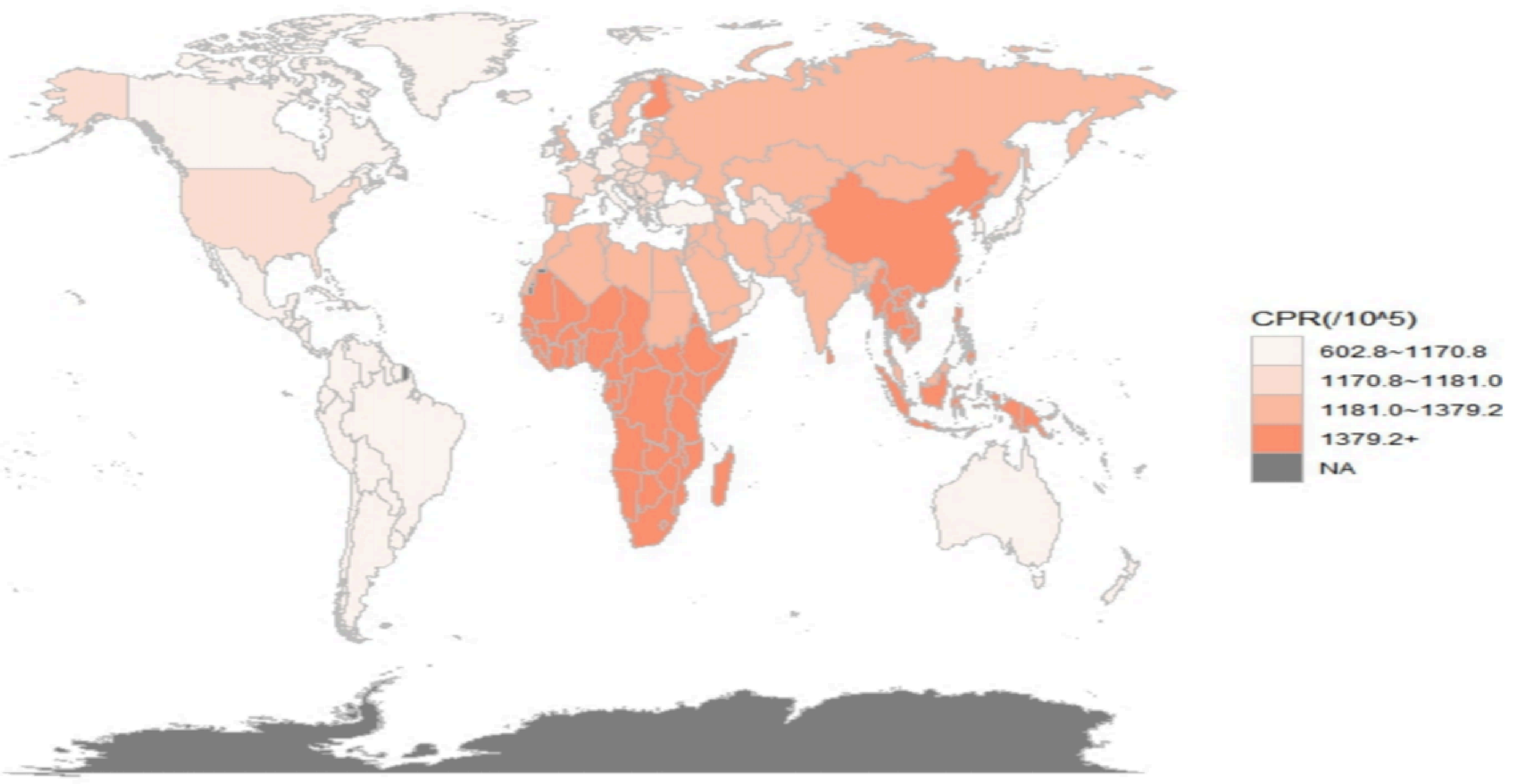
CPR(/10^5)
602.8~1170.8
1170.8~1181.0
1181.0~1379.2
1379.2+
NA

C

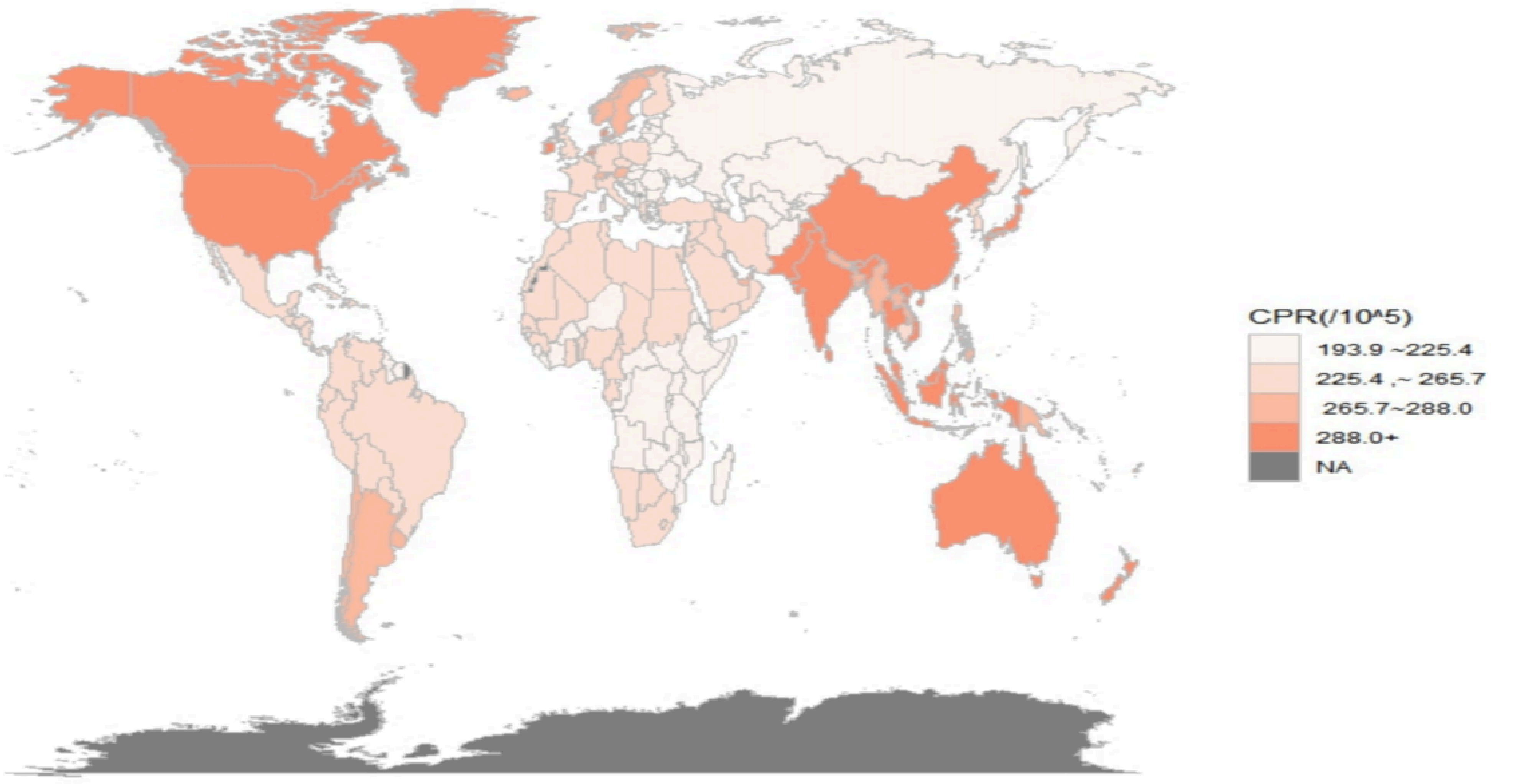
CPR(/10^5)
193.9 ~225.4
225.4 ,~ 265.7
265.7~288.0
288.0+
NA

Supplementary Figure 13: World prevalence of mental disorders 2016.(A)Anxiety disorders ;(B)Depression ;(C)Schizophrenia.

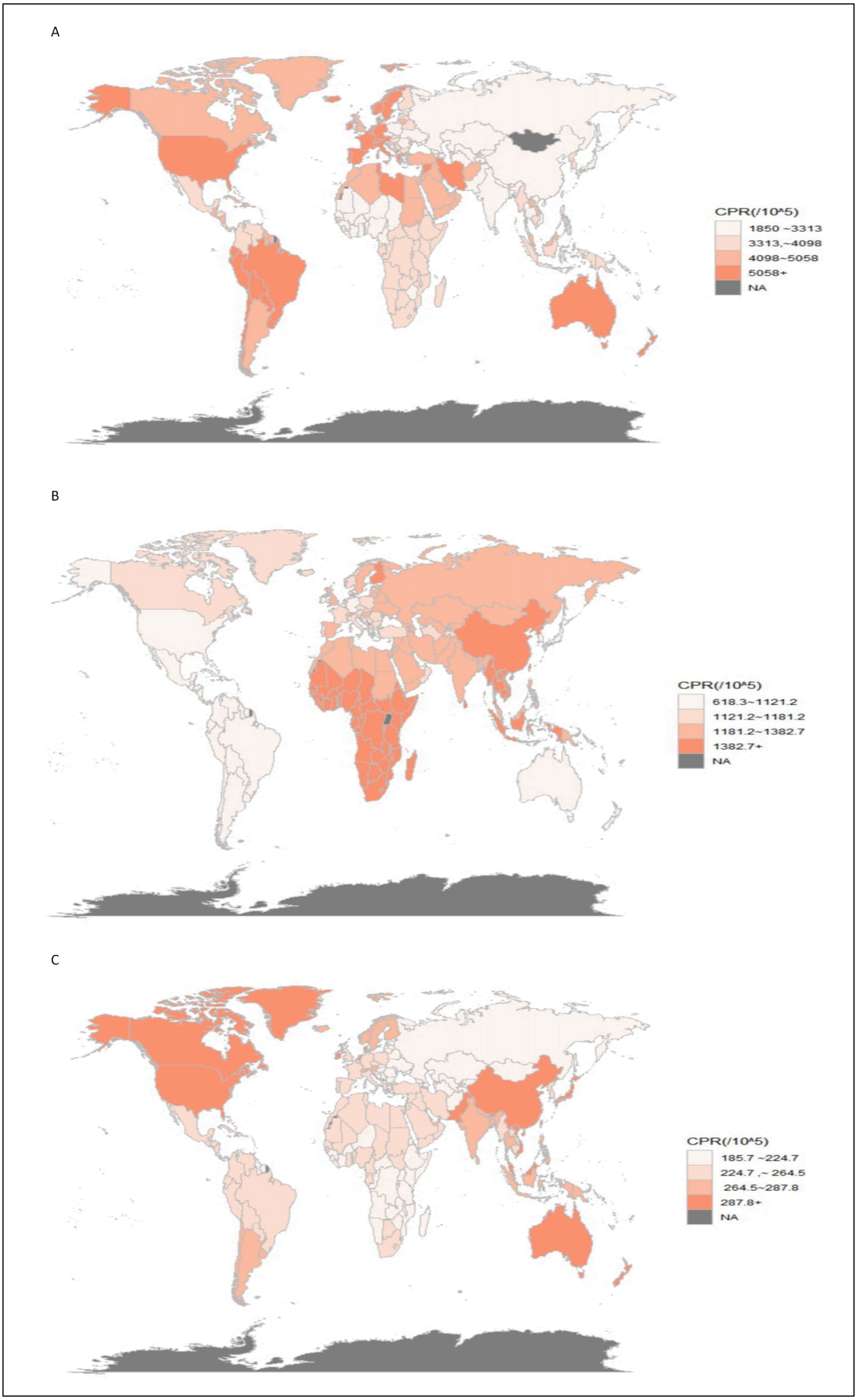
A
CPR(/10^5)
1850 ~3313
3313,~4098
4098~5058
5058+
NA
B
CPR(/10^5)
618.3~1121.2
1121.2~1181.2
1181.2~1382.7
1382.7+
NA
C
CPR(/10^5)
185.7 ~224.7
224.7 ,~ 264.5
264.5~287.8
287.8+
NA

Supplementary Figure 14: World prevalence of mental disorders 2011.(A)Anxiety disorders ;(B)Depression ;(C)Schizophrenia.
